# Efficient-tomato: a high-precision and lightweight method for cherry tomato ripeness and disease detection

**DOI:** 10.3389/fpls.2026.1880973

**Published:** 2026-10-14

**Authors:** Yunchang Zheng, Mengfan Wang, Jiacheng Sun, Leyi Han, Yunlong Ye

**Affiliations:** 1 College of Electrical Engineering, Hebei University of Architecture, Zhangjiakou, Hebei, China; 2 College of Electrical Engineering, Yanshan University, Qinhuangdao, Hebei, China; 3 The First Affiliated Hospital of Hebei North University, Zhangjiakou, Hebei, China

**Keywords:** BiFormer, CARAFE, cherry tomato, deep learning, EfficientViT, ripeness and disease detection, WIoUv1, YOLOv8s

## Abstract

Accurate detection of cherry tomatoes at different ripeness and disease stages is important for automated harvesting and crop-quality monitoring, but illumination variation, fruit overlap, leaf occlusion, complex backgrounds, and small target sizes remain challenging. This study proposes Efficient-Tomato, a lightweight detector developed from YOLOv8s through the coordinated use of EfficientViT, BiFormer, CARAFE, and WIoUv1. EfficientViT serves as the backbone for lightweight multiscale feature extraction. BiFormer is inserted into the neck to enhance informative spatial regions, while CARAFE and lightweight neck-channel adaptation improve content-aware feature reconstruction. WIoUv1 replaces the CIoU component of the bounding-box regression loss, with DFL retained. The model was evaluated on a consolidated dataset of 1,583 original images containing six categories: disease, half-ripe, overripe, ripe, rotten, and unripe. The original images were divided into training, validation, and test subsets before augmentation, and only the training subset was augmented. Each model configuration was trained independently three times, and arithmetic means were reported. Compared with YOLOv8s, Efficient-Tomato increased precision from 89.5% to 97.5%, recall from 87.3% to 96.9%, mAP@0.5 from 88.5% to 96.7%, and mAP@0.5:0.95 from 81.6% to 87.9%. The F1 score calculated from the tabulated mean precision and recall increased from 88.4% to 97.2%. FPS increased from 150.2 to 172.5, while the parameter count decreased from 22.5 M to 14.9 M. These results indicate that the coordinated design improves detection performance while reducing model complexity. Because inference speed was measured on an NVIDIA V100 GPU, further validation on embedded devices and harvesting robots is required before field deployment.

## Highlights

- A task-oriented lightweight framework for cherry tomato ripeness and disease detection.
- Complementary coordination of EfficientViT, BiFormer, CARAFE, and WIoUv1.
- Content-aware multiscale fusion improves small and occluded target detection.
- The model balances precision, inference speed, and parameter efficiency.
- The framework provides a computationally efficient basis for future evaluation on edge and robotic platforms.

## 1 Introduction

Cherry tomatoes are widely consumed because of their distinctive flavor, high yield, and relatively short ripening period. Delayed harvesting can lead to overripening, spoilage, and economic losses, whereas unripe green fruits may contain undesirable levels of solanine. Accurate detection of ripeness and disease status is therefore important for both harvesting decisions and postharvest quality control.

Tomato harvesting in China remains highly labor intensive, with labor costs accounting for approximately 50–60% of total production costs (Li, 2018). Manual harvesting is physically demanding, time consuming, and susceptible to subjective differences among workers, making it difficult to achieve consistent harvesting efficiency and ripeness standards.

Advances in artificial intelligence and robotics have promoted the development of automated perception and harvesting systems in agriculture. Robotic systems are increasingly being investigated as alternatives to repetitive manual operations and as a foundation for more autonomous production environments. Nevertheless, reliable visual perception remains difficult in unstructured agricultural scenes because illumination is uneven, fruits are frequently occluded or overlapping, and target appearance varies with ripeness and disease status. Efficient cherry tomato detection is therefore important for improving the localization accuracy and environmental adaptability of harvesting robots.

Deep learning has substantially advanced computer vision applications in agriculture, particularly crop detection and recognition. Earlier object-detection methods relied on manually designed representations followed by conventional classifiers. Representative approaches include Haar-like features (Viola and Jones, 2004), Histograms of Oriented Gradients (HOG) (Tan et al., 2020), and Deformable Part Models (DPM) (Felzenszwalb et al., 2010).

These traditional methods depend strongly on handcrafted features and often exhibit limited robustness and generalization in complex scenes. Deep-learning detectors instead learn hierarchical representations directly from data and are generally divided into two-stage and one-stage frameworks. Two-stage detectors first generate candidate regions and then classify and refine them. Representative methods include the Region-based Convolutional Neural Network (R-CNN) (Girshick et al., 2014), Fast R-CNN (Girshick, 2015), Faster R-CNN (Ren et al., 2015), and Mask R-CNN (He et al., 2017). Although these detectors often provide strong localization performance, their multi-stage inference pipelines can limit processing speed.

One-stage detectors directly predict object classes and bounding boxes in a single forward pass, providing a more favorable balance between accuracy and speed for agricultural applications. Representative models include the You Only Look Once (YOLO) family (Redmon et al., 2016; Redmon and Farhadi, 2017, Redmon and Farhadi, 2018; Bochkovskiy et al., 2020; Jocher et al., 2022; Li et al., 2022; Wang et al., 2023), the Single Shot MultiBox Detector (SSD) and its variants (Liu et al., 2016; Jeong et al., 2017; Li et al., 2017; Fu et al., 2017), and EfficientDet (Tan et al., 2020). Transformer-based detection has also emerged as an important direction. The Detection Transformer (DETR) (Carion et al., 2020), for example, introduced the Transformer architecture (Vaswani et al., 2017) into end-to-end object detection and provided an alternative to conventional convolutional detection pipelines.

A range of machine-vision and deep-learning methods has been investigated for tomato and fruit detection. Chen and Yang (Chen and Yang, 2013) developed a practical vision-based approach for ripe tomato recognition and localization, demonstrating the potential of machine vision for automated harvesting. Zhao et al (Zhao et al., 2016). combined an AdaBoost classifier with color analysis and Haar-like features for tomato detection. Although the method achieved reasonable detection performance, its processing speed was insufficient for real-time robotic harvesting. Sa et al (Sa et al., 2016). applied Faster R-CNN to fruit detection and fused red, green, and blue (RGB) and near-infrared information using two strategies. The method outperformed earlier approaches, but the detection of small fruits and inference efficiency remained limited. Yuan et al (Yuan et al., 2020). developed an SSD-based method for robust cherry tomato detection in greenhouse environments, while further improvements in computational efficiency and small-target detection remained important for robotic harvesting applications. Fatchurrahman et al (Fatchurrahman et al., 2020). used fluorescence imaging to discriminate between mature-green and immature-green tomatoes, showing that optical characteristics can provide useful information for maturity assessment. Su et al (Su et al., 2022). developed a lightweight YOLOv3 model with a MobileNetV1 backbone for tomato-ripeness classification and reported an average accuracy of 97.5%, including under partial leaf occlusion. Li et al (Li et al., 2023). proposed a YOLOv5s-based tomato-ripeness model with a reported average accuracy of 97.42%, with particular emphasis on improving the recognition of occluded small targets.

Mu et al (Mu et al., 2020). developed a greenhouse tomato-detection framework based on Faster R-CNN and ResNet-101 pretrained on the Common Objects in Context (COCO) dataset, obtaining an accuracy of 87.83%. Sun et al (Sun et al., 2020). incorporated a feature pyramid network into a convolutional neural network (CNN)-based detector and reported an increase in detection rate from 90.7% to 99.5% relative to a conventional Faster R-CNN model. Liu et al (Liu et al., 2020). proposed YOLO-Tomato by integrating dense feature extraction and a customized bounding-box representation into YOLOv3. The model achieved a detection rate of 94.58% under moderate occlusion, approximately four percentage points higher than under heavy occlusion.

Yue et al (Yue et al., 2023). developed an improved YOLOv8-Seg network for instance segmentation of healthy and diseased tomato plants during the growth stage. The method achieved a mean average precision at an Intersection over Union threshold of 0.5 (mAP@0.5) of 92.2%, with a reported inference time of 3.5 milliseconds (ms). In June 2023, Yang et al (Yang et al., 2023). put forward a lightweight YOLOv8 tomato detection algorithm integrating functional enhancement and attention mechanisms. The enhanced YOLOv8s network exhibits a reduced loss and reaches an mAP@0.5 of 93.4% on the given dataset. Compared with the baseline model, mAP@0.5 increased by 1.5 percentage points, precision by 2.0 percentage points, and recall by 0.8 percentage points.

Recent studies have increasingly emphasized lightweight architecture design, multi-category ripeness recognition, Transformer-based feature modeling, and practical edge deployment. Zhang et al. (2023) divided cherry tomatoes into four occlusion-related categories and developed an improved YOLOv4-Tiny detector for daytime and nighttime harvesting scenes. Wang et al. (2024) proposed GreenFruitDetector, a lightweight YOLOv8-based model that combines multiscale feature enhancement with channel pruning for green-fruit detection under occlusion and fruit–leaf color similarity. Liu et al. (2024) combined a lightweight YOLOv7 detector with HRNet to segment greenhouse cherry tomatoes at four maturity levels. Sun et al. (2025) developed a lightweight YOLOv8-based model for four-stage tomato-ripeness detection and deployed an application on an Android platform. For tomato pest and disease detection, Yang et al. (2025) integrated MobileNetV3 with a streamlined Swin Transformer and evaluated the resulting lightweight model on an NVIDIA Jetson Orin NX edge platform. More recently, Yang et al. (2026) proposed a lightweight RT-DETR-based detector for on-device truss tomato detection and reported deployment on an NVIDIA Jetson Orin Nano Super.

Nevertheless, as depicted in **Figure 1**, cherry tomato detection in natural agricultural environments remains challenging. Illumination variation, occlusion, fruit overlap, color similarity between unripe fruits and leaves, and a high proportion of small targets can reduce classification and localization performance. Existing high-capacity detectors may also impose substantial computational and storage requirements, which limits their potential use on resource-constrained agricultural platforms. Existing multi-category cherry tomato studies have mainly defined classes according to occlusion conditions or ripeness stages. For example, Zhang et al. (2023) distinguished four occlusion-related categories, whereas Liu et al. (2024) considered four maturity levels. Recent lightweight studies have also generally treated ripeness detection, fruit detection, or pest and disease detection as separate tasks (Sun et al., 2025; Yang et al., 2025, Yang et al., 2026). However, comparatively few studies have jointly represented normal ripeness stages and abnormal fruit conditions within a single object-detection framework. The combined detection of disease, half-ripe, overripe, ripe, rotten, and unripe cherry tomatoes therefore remains insufficiently investigated.

**Figure 1:**
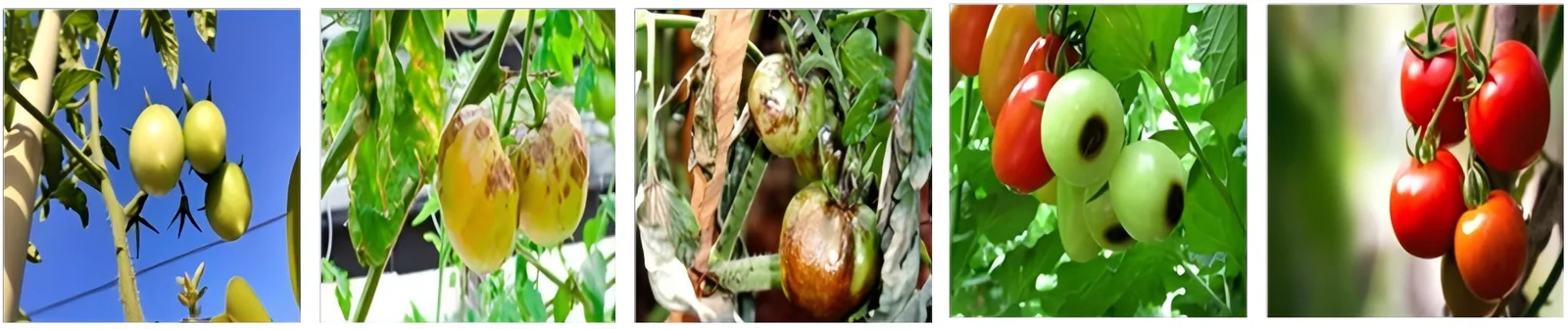
Representative cherry tomato images.

To address these challenges, this study develops Efficient-Tomato from the YOLOv8s baseline. The scientific contribution does not arise from proposing a new individual operator. Instead, it lies in the task-oriented coordination of lightweight feature extraction, sparse attention-based feature enhancement, content-aware multiscale fusion, and bounding-box regression optimization. These components are placed at different stages of the detection pipeline and are designed to address complementary limitations rather than provide redundant feature enhancement.

The main contributions of this study are as follows:

1. A consolidated six-category dataset is organized from field-collected and publicly available images, covering disease, half-ripe, overripe, ripe, rotten, and unripe targets under illumination variation, occlusion, overlap, complex backgrounds, and scale variation. The original images are partitioned before augmentation to prevent transformed versions of the same image from appearing in different subsets.
2. A task-oriented lightweight detection framework is developed by coordinating four existing components at different stages of YOLOv8s. Efficient Vision Transformer (EfficientViT) reduces backbone complexity, BiFormer strengthens selective spatial feature representation through bi-level routing attention, Content-Aware ReAssembly of FEatures (CARAFE) is integrated with lightweight neck channel adaptation to improve content-aware multiscale reconstruction while controlling complexity, and Wise-IoU version 1 (WIoUv1) refines bounding-box regression. Their complementary roles are explicitly analyzed in the methodology section.
3. Ablation and comparative experiments quantify the contribution of each component and evaluate the balance among precision, recall, mean average precision (mAP), F1 score, inference speed in frames per second (FPS), and model size. The results show that Efficient-Tomato improves small-target and occlusion-related detection while reducing the parameter count relative to YOLOv8s.

The remainder of this study is organized as follows. Section 2 describes the YOLOv8s baseline, the proposed Efficient-Tomato framework, and the complementary roles of its components. Section 3 presents the dataset, experimental settings, evaluation metrics, ablation experiments, and comparative results. Section 4 summarizes the main findings, limitations, and future research directions.

## 2 Methodology

### 2.1 YOLOv8s baseline

YOLOv8s was selected as the baseline because it provides a practical balance among detection performance, inference speed, and model size. Its architecture consists of a backbone for feature extraction, a neck for multiscale feature fusion, and a decoupled detection head for classification and bounding-box regression.

The standard backbone uses convolutional layers, Cross Stage Partial bottleneck modules with two convolutions (C2f), and a Spatial Pyramid Pooling–Fast (SPPF) module. The C2f module improves gradient flow and feature reuse while limiting computational cost. The SPPF module is a streamlined implementation derived from the spatial pyramid pooling concept (He et al., 2015). It applies sequential max-pooling operations to enlarge the receptive field and aggregate multiscale contextual information with relatively low computational cost.

The neck adopts a bidirectional multiscale feature-fusion design with top-down and bottom-up pathways, following the path-aggregation concept (Liu et al., 2018). The top-down pathway transfers high-level semantic information to higher-resolution features, whereas the bottom-up pathway strengthens localization information. This bidirectional fusion supports the detection of objects at different scales.

YOLOv8s uses an anchor-free decoupled head. Binary cross-entropy loss is used for classification, and Distribution Focal Loss (DFL) (Li et al., 2020) together with Complete Intersection over Union (CIoU) loss (Zheng et al., 2020) is used for bounding-box regression. A task-aligned assignment strategy (Feng et al., 2021) dynamically selects positive samples during training.

### 2.2 Proposed efficient-tomato framework

Although YOLOv8s provides a favorable balance between detection performance and model complexity, its performance can degrade when cherry tomatoes occupy small image regions, overlap with adjacent fruits, are partially occluded by leaves, or exhibit colors similar to the background. In such cases, lightweight feature extraction may weaken fine-grained information, conventional feature fusion may inadequately reconstruct object boundaries, and bounding-box regression may be sensitive to samples of varying localization quality.

Efficient-Tomato addresses these challenges through a coordinated modification of the backbone, neck, upsampling operation, and regression loss. EfficientViT is used as the backbone to reduce computational and parameter costs while retaining local and global feature extraction. BiFormer is inserted into the neck to select informative spatial regions and strengthen contextual dependencies for small and occluded targets. CARAFE replaces nearest-neighbor upsampling, and the channel configuration of the adjacent neck feature-fusion layers is lightly adapted to align input and output dimensions and control the additional computational cost. Finally, WIoUv1 replaces the CIoU component of the bounding-box regression loss, whereas DFL is retained.

These components were selected according to the visual characteristics of cherry tomato detection rather than introduced as a generic module combination. Cherry tomatoes frequently appear as small, densely distributed, overlapping, and partially leaf-occluded targets, while adjacent ripeness and disease categories may exhibit only subtle differences in color, texture, and boundary appearance. EfficientViT is therefore suitable for reducing backbone complexity while retaining local details and broader contextual information. BiFormer further emphasizes task-relevant regions and suppresses redundant responses from leaves, stems, support structures, and other greenhouse backgrounds, which is beneficial when targets are partially occluded or visually similar to their surroundings. During multiscale fusion, CARAFE reconstructs high-resolution features using content-dependent reassembly kernels, helping to preserve the boundaries and fine-grained appearance cues of small or closely adjacent fruits. WIoUv1 complements these feature-level modifications at the optimization stage by improving bounding-box localization for samples with different localization quality. Accordingly, the substantive contribution of Efficient-Tomato lies in the task-oriented coordination of these complementary mechanisms for the specific difficulties of cherry tomato ripeness and disease detection, rather than in claiming novelty for any individual module.

The proposed framework should therefore be understood as a task-oriented system design rather than a simple collection of independent modules. EfficientViT establishes the lightweight computational basis; BiFormer compensates for potential feature degradation by emphasizing informative regions; CARAFE improves the transfer of fine-grained information during feature fusion; and WIoUv1 refines object localization during optimization. The design rationale and complementary relationships among these components are discussed in Section 2.2.5.

#### 2.2.1 EfficientViT backbone

Conventional convolutional backbones can provide strong feature representations but may impose substantial computational and memory costs. To reduce the complexity of YOLOv8s while preserving multiscale information, EfficientViT (Cai et al., 2022) is used as the backbone of Efficient-Tomato. EfficientViT combines efficient convolutional operations with lightweight multiscale attention, enabling the network to model local details and long-range contextual relationships with reduced computational overhead.

No external pretrained weights were used in this study. The EfficientViT backbone and the remaining detection network were randomly initialized and trained end-to-end from scratch on the cherry tomato dataset. No pretrained parameters from ImageNet, COCO, or any other external dataset were used.

The general Vision Transformer concept introduced by Dosovitskiy et al (Dosovitskiy et al., 2020). divides an input image into patches, which are projected into token embeddings and processed by Transformer encoders. In dense prediction tasks, direct global self-attention can be computationally expensive at high spatial resolutions. EfficientViT addresses this issue through efficient attention and convolutional building blocks that are more suitable for high-resolution feature extraction.

The EfficientViT backbone contains mobile inverted bottleneck convolution (MBConv) blocks and EfficientViT blocks. MBConv uses pointwise expansion, depthwise convolution, and pointwise projection to extract local features with limited computational cost (Sandler et al., 2018). The EfficientViT block combines MBConv with lightweight multiscale attention to integrate local image details and global contextual information.

The lightweight multiscale attention module processes features at multiple spatial scales and fuses the resulting representations along the channel dimension. Local convolutional branches provide fine-grained information, while the attention branches capture broader dependencies. This combination is beneficial for cherry tomato images because color, texture, and boundary cues must be retained for small, overlapping, and partially occluded objects.

In Efficient-Tomato, the EfficientViT backbone provides multiscale feature maps for the neck. Its role is not only to reduce the parameter count but also to establish a lightweight feature-extraction basis for the subsequent BiFormer and CARAFE modules.

#### 2.2.2 BiFormer attention in the neck

Standard self-attention evaluates relationships among a large number of token pairs, including many pairs that are weakly related to the target. BiFormer (Zhu et al., 2023) reduces this redundancy through Bi-Level Routing Attention (BRA). BRA first identifies relevant regions at a coarse level and then performs token-level attention only within the selected regions. This dynamic sparse routing enables the network to preserve long-range dependencies while avoiding unnecessary attention computation.

As shown in **Figure 2**, a BiFormer block consists of a 3×3 depthwise convolution for positional encoding, a BRA module for spatial interaction, and a multilayer perceptron for channel transformation. Residual connections and layer normalization are used to support stable feature learning.

**Figure 2:**
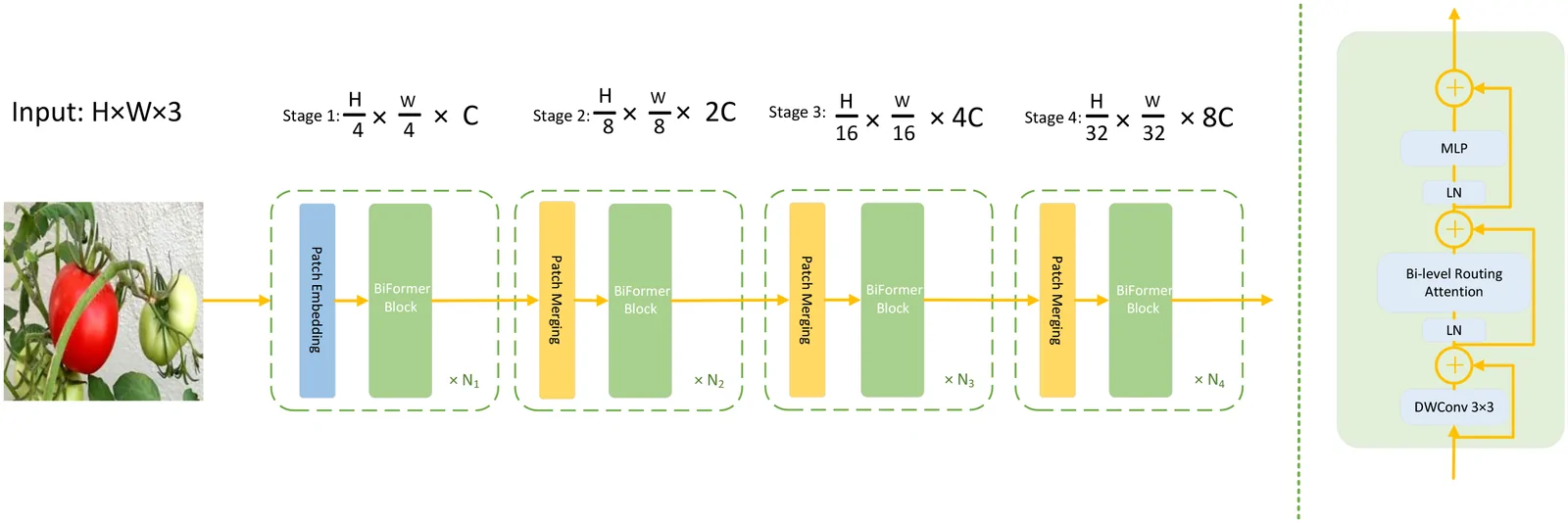
Overall BiFormer architecture and detailed structure of a BiFormer block.

The routing process of BRA is illustrated in **Figure 3**. Query and key representations are first summarized at the region level to determine the most relevant routing regions. Fine-grained attention is then calculated between tokens in the selected regions. This mechanism allows the model to emphasize cherry tomato targets and suppress irrelevant leaves, stems, and background areas.

**Figure 3:**
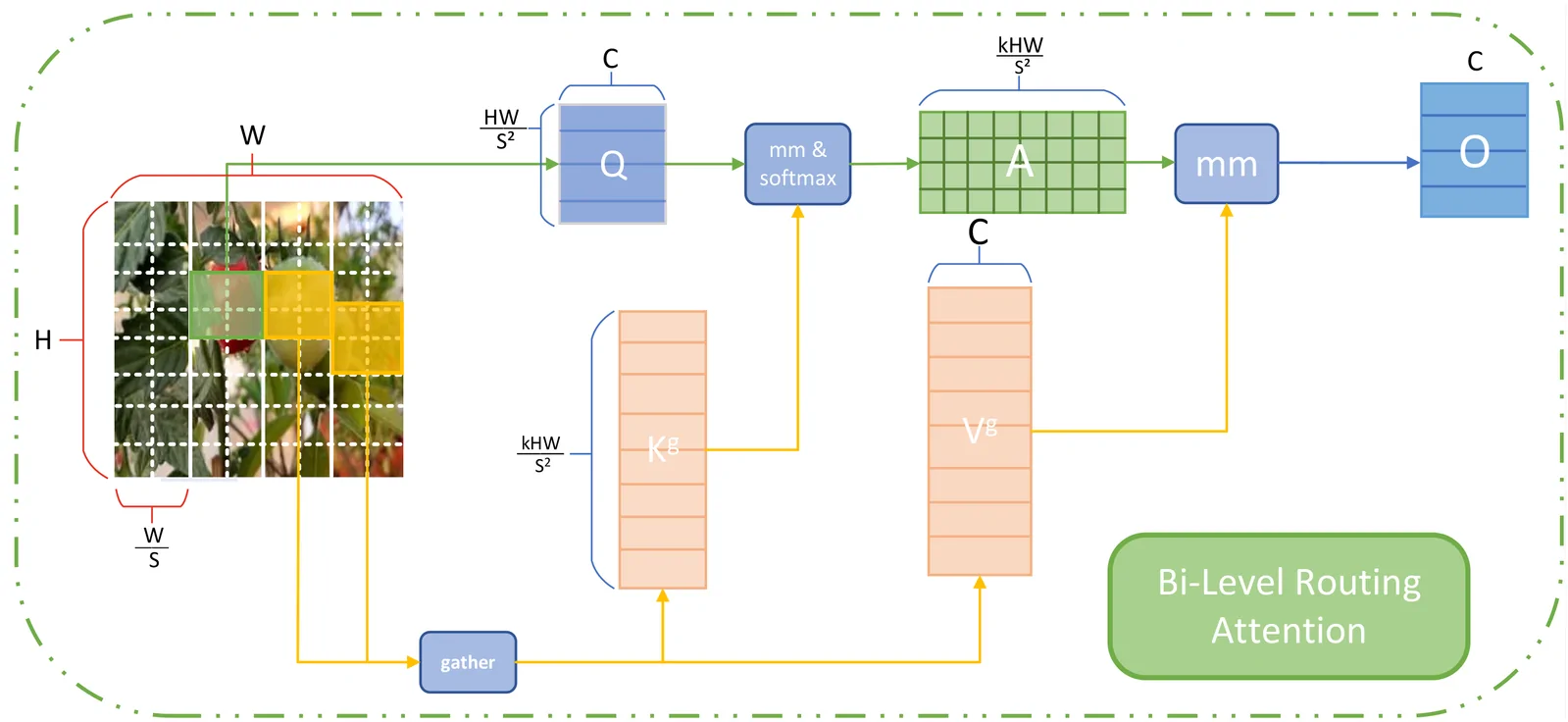
Structure of Bi-Level routing attention.

In the implemented neck, BiFormer modules are placed at the P3, P4, and P5 multiscale feature-fusion positions. For each BRA module, the input feature map is partitioned into a 7×7 grid of regions. Each query region selects the four most relevant regions for fine-grained token-level attention, and the routing parameter is therefore set to top-*k* = 4. The number of attention heads is set to 8, while the projection dimensions of the query and key are kept equal to the corresponding input-channel dimension. The multilayer-perceptron expansion ratio is set to 4. Positional encoding is implemented using a 3×3 depthwise convolution, and both attention dropout and projection dropout are set to 0. Non-parametric hard routing is adopted, without differentiable routing or soft routing. These settings are kept unchanged in all ablation and comparative experiments. Consistent with the BRA mechanism, region-level relevance selection is performed first, followed by token-level attention only within the selected regions.

BiFormer modules are integrated into the neck of Efficient-Tomato, where features from different backbone stages are fused. Their primary role is to compensate for potential information loss caused by lightweight feature extraction and to strengthen the representation of small, overlapping, and partially occluded cherry tomatoes.

#### 2.2.3 CARAFE content-aware upsampling

The standard YOLOv8s neck uses nearest-neighbor interpolation during top-down feature fusion. This operation depends only on spatial position and applies the same fixed rule to all feature content, which may limit the reconstruction of object boundaries and fine-grained textures. CARAFE (Wang et al., 2019) replaces this operation with content-aware feature reassembly.

As shown in **Figure 4**, CARAFE consists of an upsampling-kernel prediction module and a content-aware reassembly module. The kernel prediction module first compresses the channels of the input feature map and then predicts a reassembly kernel for each spatial location. A softmax operation normalizes the kernel weights. The reassembly module uses these content-dependent kernels to aggregate features within a relatively large receptive field.

**Figure 4:**
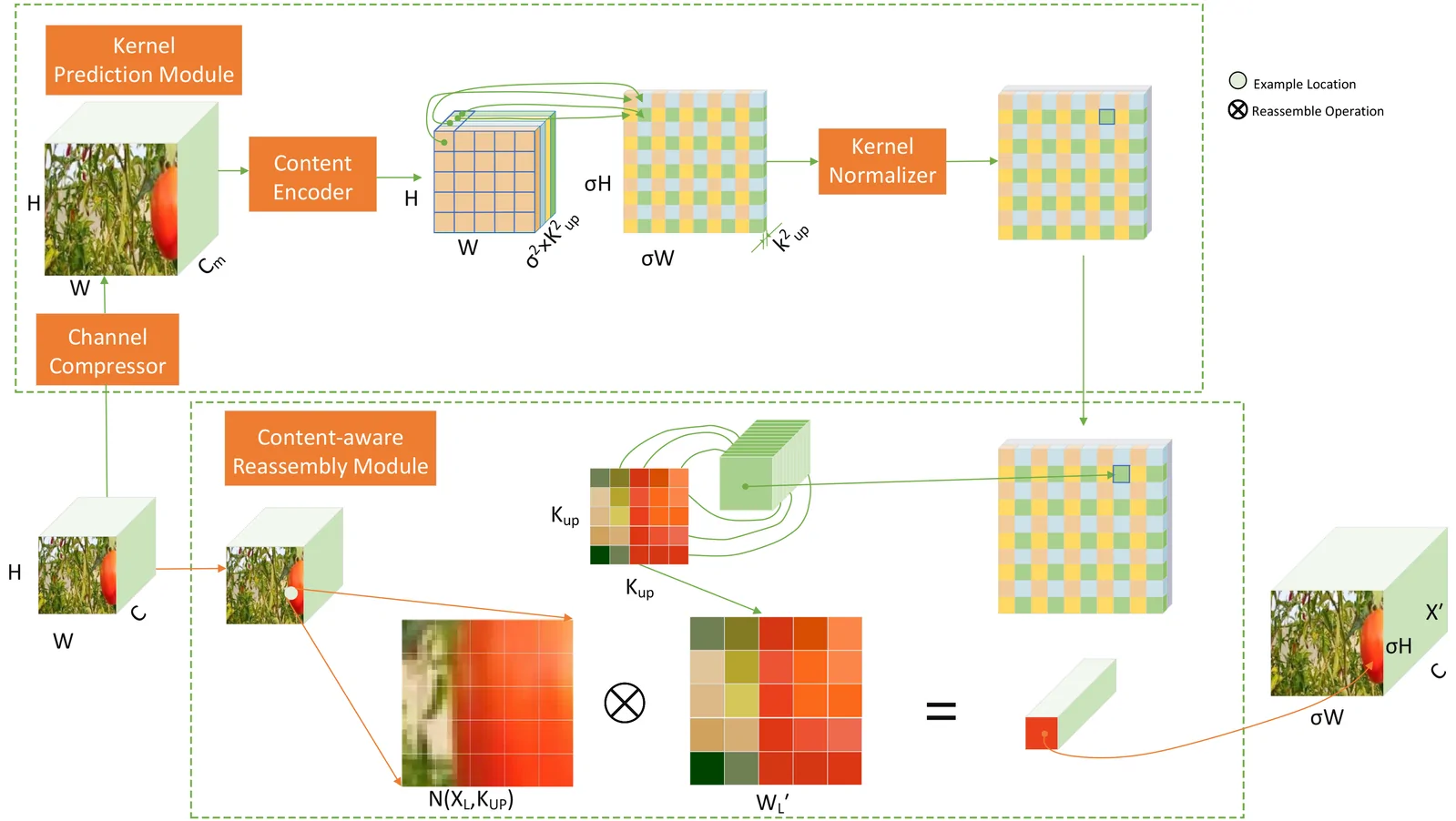
General framework of CARAFE, including kernel prediction and content-aware feature reassembly.

In this study, the compressed channel number *C_m_* is set to 64, the predicted upsampling-kernel size *K*_up_ is set to 5, and the encoder-kernel size *K*_encoder_ is set to 3. When CARAFE is integrated into the neck, the channel widths of the adjacent feature-fusion layers are correspondingly adapted to match the input and output feature dimensions and to control the additional computational overhead. Conceptually, this integration contains two distinct changes. The first is a lightweight channel-width adaptation of the adjacent fusion layers, which primarily changes feature dimensions and model complexity while leaving the upsampling rule itself unchanged. The second is the replacement of nearest-neighbor interpolation by CARAFE under the adapted channel configuration, which changes how high-resolution features are reconstructed from local content. Therefore, the configuration used in the main progressive ablation represents the joint effect of channel adaptation and content-aware upsampling rather than a strict single-module replacement. BiFormer selects and enhances informative regions, whereas the CARAFE integration configuration is intended to preserve and reconstruct their boundary, color, and texture information during multiscale fusion.

#### 2.2.4 WIoUv1 bounding-box regression loss

Cherry tomato images contain numerous small, overlapping, and partially occluded objects, which can produce bounding boxes with varying localization quality. YOLOv8s uses Complete Intersection over Union (CIoU) (Zheng et al., 2020) together with Distribution Focal Loss (DFL) for bounding-box regression. CIoU considers overlap, center-point distance, and aspect-ratio consistency between the predicted and ground-truth boxes. Its formulation and loss are expressed in Equations 1 and Equations 2, respectively.

(1) $$CIoU=IoU-\frac{\rho^{2}\left(b,b^{gt}\right)}{c^{2}}-\alpha \nu$$

(2) $$L_{CIoU}=1-IoU+\frac{\rho^{2}\left(b,b^{gt}\right)}{c^{2}}+\alpha \nu$$

Here, IoU denotes the intersection-over-union between the predicted and ground-truth boxes; *b* and *b^gt^* denote their center points; *c* is the diagonal length of the smallest enclosing box; and *αν* measures aspect-ratio consistency.

In Efficient-Tomato, WIoUv1 (Tong et al., 2023) replaces the CIoU component of the bounding-box regression loss, while DFL is retained. WIoUv1 introduces a distance-based attention factor that adjusts the contribution of the IoU loss according to the center-point distance between the predicted and ground-truth boxes. Its formulation is given in Equations 3 and Equations 4:

(3) $$L_{\text{WIoUv}1}=R_{\text{WIoU}}L_{\text{IoU}}$$

(4) $$R_{\text{WIoU}}=\exp\left(\frac{{\left(x-x_{gt}\right)}^{2}+{\left(y-y_{gt}\right)}^{2}}{W_{g}^{2}+H_{g}^{2}}\right),$$

where (*x*, *y*) and (*x*_gt_, *y*_gt_) are the center coordinates of the predicted and ground-truth boxes, respectively, and *W_g_* and *H_g_* are the width and height of their smallest enclosing box. This formulation increases sensitivity to center-point displacement without introducing an additional aspect-ratio penalty. WIoUv1 is used only as a training-stage bounding-box regression loss; it contains no learnable parameters and does not alter the inference network. Accordingly, its contribution is evaluated in terms of localization optimization and detection accuracy rather than model compression or inference acceleration. In the proposed framework, WIoUv1 complements the feature-level modifications by improving localization optimization for small, overlapping, and partially occluded cherry tomatoes. **Figure 5** illustrates the geometric relationships used by CIoU and WIoUv1.

**Figure 5:**
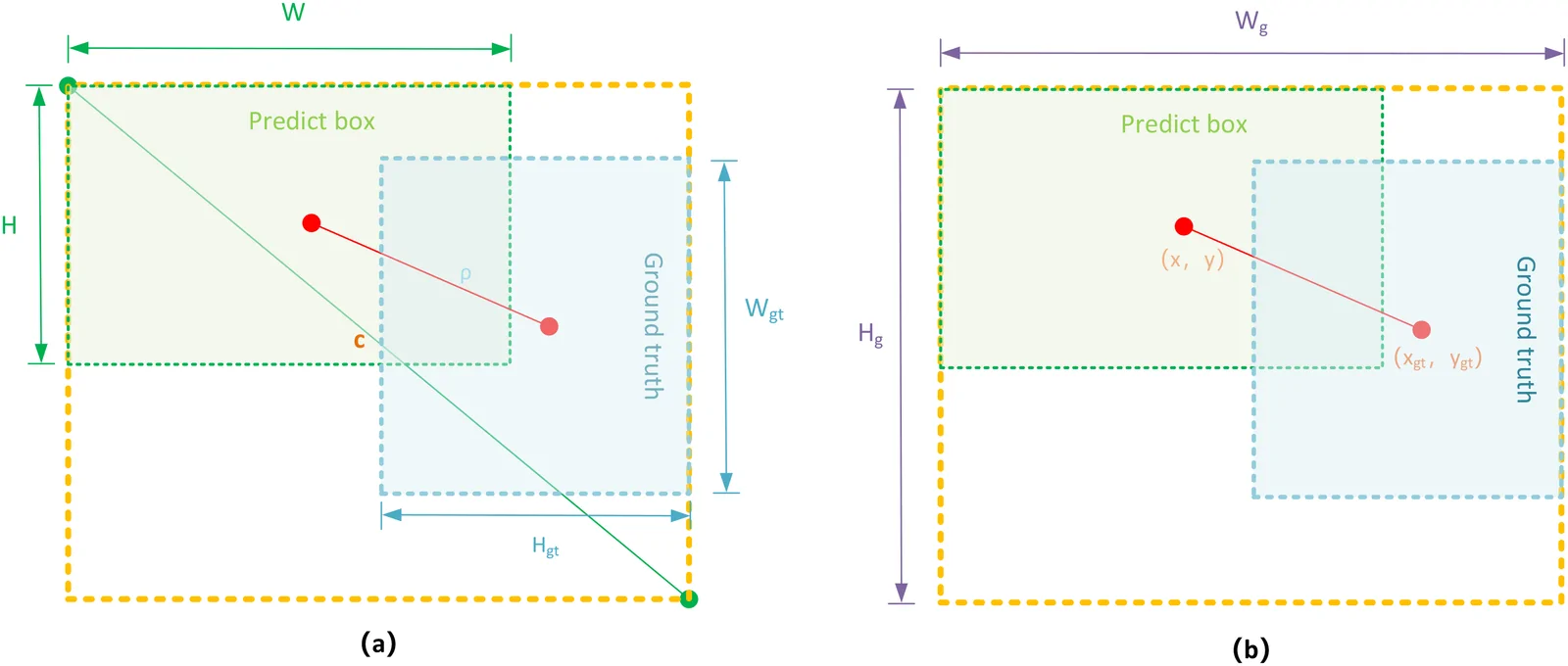
Geometric relationships used in bounding-box regression: **(a)** CIoU; **(b)** WIoUv1.

#### 2.2.5 Complementarity and design rationale

The novelty of Efficient-Tomato lies in coordinating existing components according to the characteristics of the cherry tomato detection task rather than presenting any individual component as a newly proposed operator. As summarized in **Table 1**, the four components operate at different stages of the detection pipeline and address distinct but related limitations.

**Table 1:** Complementary roles of the components in efficient-tomato.

Component	Location	Primary role	Complementary contribution
EfficientViT	Backbone	Lightweight local and global feature extraction	Establishes the computational basis for the framework
BiFormer	Neck	Selective spatial and contextual feature enhancement	Compensates for feature loss and suppresses background redundancy
CARAFE	Feature fusion	Content-aware upsampling and detail reconstruction	Preserves boundaries and textures of small or overlapping targets
WIoUv1	Regression loss	Center-distance-aware bounding-box optimization	Improves localization after feature extraction and fusion

EfficientViT first reduces backbone complexity, but aggressive lightweight design may weaken the representation of small or partially occluded objects. BiFormer addresses this potential limitation by selecting informative regions and modeling contextual relationships in the neck. CARAFE then reconstructs the selected multiscale features using content-dependent kernels, thereby preserving boundaries, color transitions, and texture cues that are important for distinguishing adjacent ripeness and disease categories. WIoUv1 acts at the optimization stage and improves the alignment of predicted boxes with ground-truth objects. Consequently, the four components form a sequential and complementary pipeline spanning feature extraction, feature selection, feature reconstruction, and localization optimization.

#### 2.2.6 Efficient-tomato architecture

The overall architecture of Efficient-Tomato is shown in **Figure 6**. EfficientViT replaces the original YOLOv8s backbone and outputs multiscale feature maps for subsequent fusion. BiFormer modules are integrated into the neck to enhance informative spatial features and contextual relationships. CARAFE is used in place of nearest-neighbor upsampling to perform content-aware feature reconstruction, with lightweight channel adaptation applied to the adjacent neck fusion layers. In the detection head, binary cross-entropy loss is used for classification, while WIoUv1 and DFL are used for bounding-box regression. Through this coordinated design, Efficient-Tomato aims to improve the detection of disease, half-ripe, overripe, ripe, rotten, and unripe cherry tomatoes while maintaining a lightweight model structure. 

**Figure 6:**
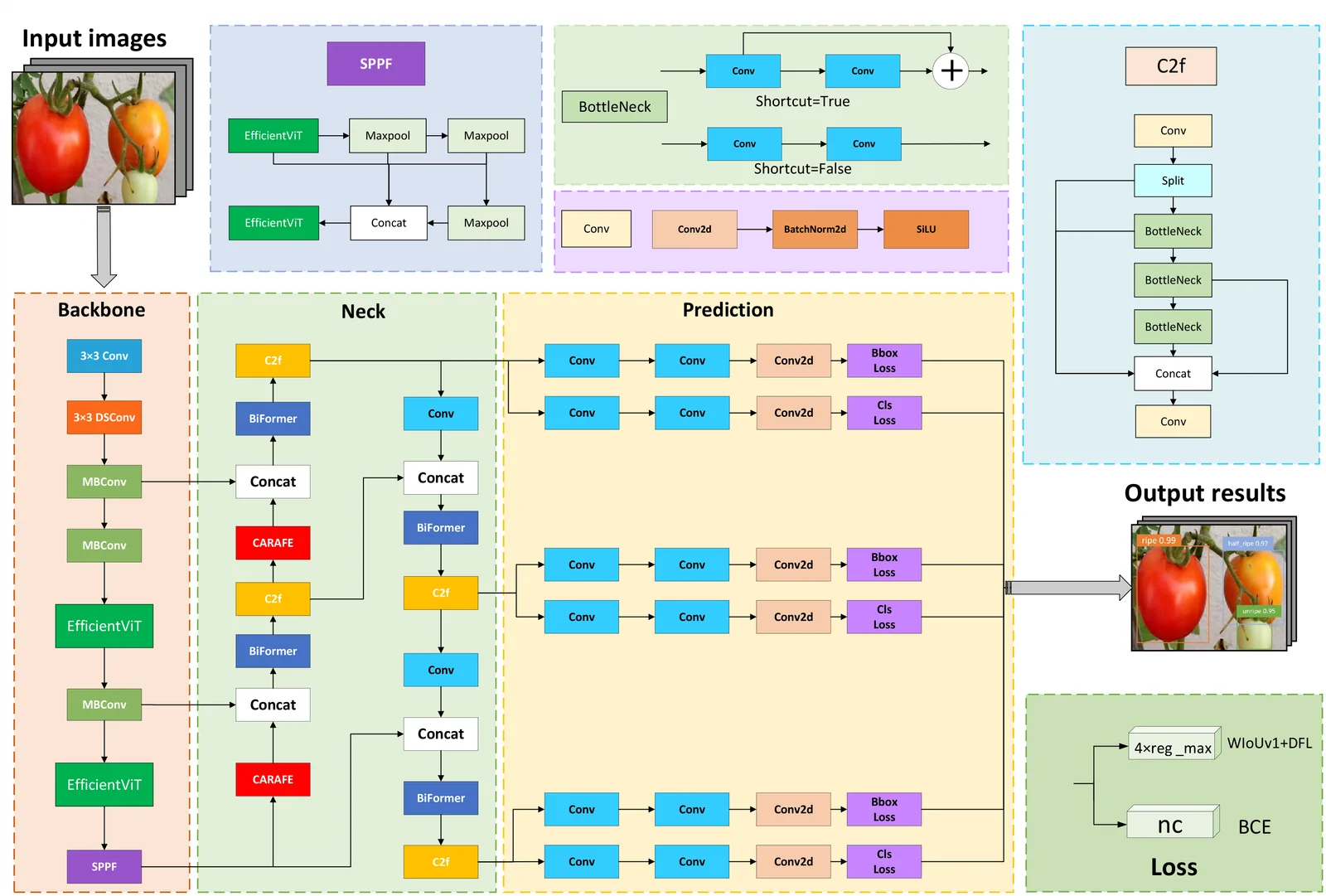
The Efficient-Tomato framework.

## 3 Experiment and discussion

### 3.1 Dataset and preprocessing

The overall experimental workflow of Efficient-Tomato is shown in **Figure 7**. The dataset used in this study was consolidated from field-collected images and publicly available images. A total of 682 cherry tomato images were collected at the Dazhouzhuang cherry tomato production base in Changli County, Qinhuangdao, Hebei Province, China, and an additional 901 images were selected from the publicly available Cherry Tomatoes Dataset on Roboflow Universe (Drapes, 2023). Together, these sources yielded 1,583 original images. Field acquisition was conducted mainly during the cherry tomato growth and ripening period from June to August 2023, primarily between 08:00 and 17:00. Most images were captured inside production greenhouses under natural ambient illumination. Background elements encountered in actual production environments, including leaves, support structures, stems, and soil, were intentionally retained.

**Figure 7:**
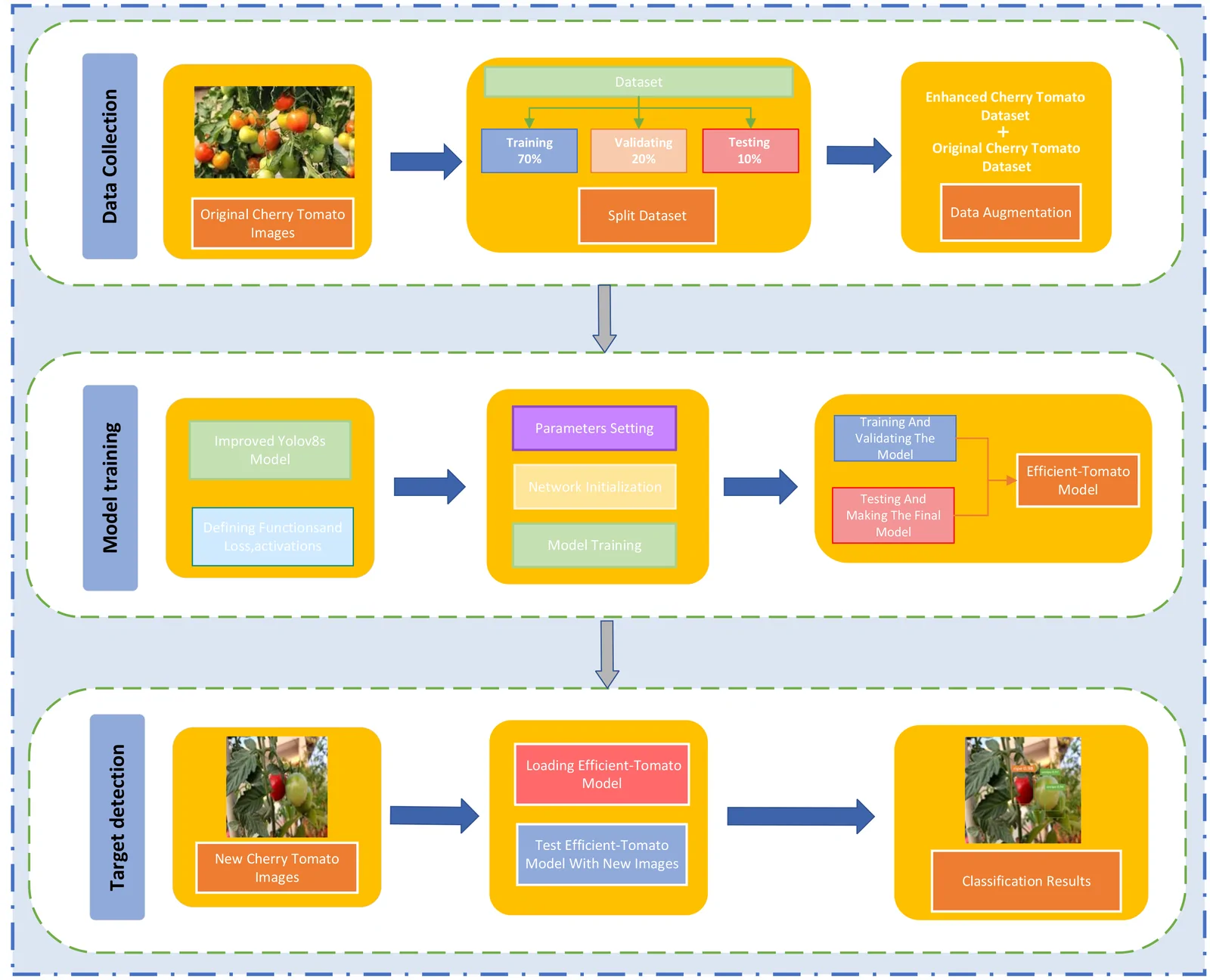
Overall experimental workflow of Efficient-Tomato.

The field images were mainly captured using the rear camera of a smartphone with an approximate resolution of 12 megapixels. Most original images had a landscape resolution of 4032×3024 pixels, while some portrait-oriented images had a resolution of 3024×4032 pixels. The acquisition process covered different shooting distances, observation angles, and illumination conditions, including dense fruit clusters, fruit overlap, leaf occlusion, local shadows, complex backgrounds, and substantial target-scale variation.

The original labels associated with the public images were not used directly. Both the 682 field-collected images and the 901 images selected from Roboflow were manually re-annotated using LabelImg according to the same category definitions and bounding-box rules. All visible targets were assigned to one of six categories: disease, half-ripe, overripe, ripe, rotten, and unripe.

To reduce ambiguity among visually adjacent categories, annotation was based on the dominant visible appearance of each fruit. Unripe fruits were defined as predominantly green fruits without obvious abnormal surface damage; half-ripe fruits showed a clear transitional mixture of green, yellow/orange, and red coloration; ripe fruits were predominantly red and visually intact; and overripe fruits showed darker or excessively mature coloration and visible aging-related appearance without clear extensive decay. The disease category was used for fruits showing localized lesions, abnormal spots, discoloration, or surface irregularities inconsistent with normal ripening, whereas the rotten category was reserved for fruits with obvious extensive decay, dark necrotic regions, tissue collapse, or decomposition. When an abnormal condition was visually evident, the fruit was assigned according to its dominant visible appearance. Disease and rotten samples were distinguished using the above morphological criteria rather than a fixed priority rule; borderline cases between these two categories were re-examined during annotation review. Bounding boxes were drawn as tightly as practical around the visible fruit extent while avoiding inclusion of neighboring fruits and unrelated background regions. Partially occluded fruits were annotated when sufficient visible evidence remained to determine both the fruit boundary and its category.

After the initial annotation, borderline samples, category assignments, and bounding-box extents were jointly reviewed for consistency. Ambiguous cases were re-examined against the same operational criteria, and only annotations for which a consistent label could be assigned were retained. This review was particularly important for the boundaries between half-ripe and ripe, ripe and overripe, and disease and rotten. The annotations were initially stored in Extensible Markup Language (XML) files following the Pattern Analysis, Statistical Modelling and Computational Learning Visual Object Classes (PASCAL VOC) format and were subsequently converted into the format required for YOLO model training. Examples of the annotation process and the six cherry tomato categories are shown in **Figures 8** and **9**, respectively.

**Figure 8:**
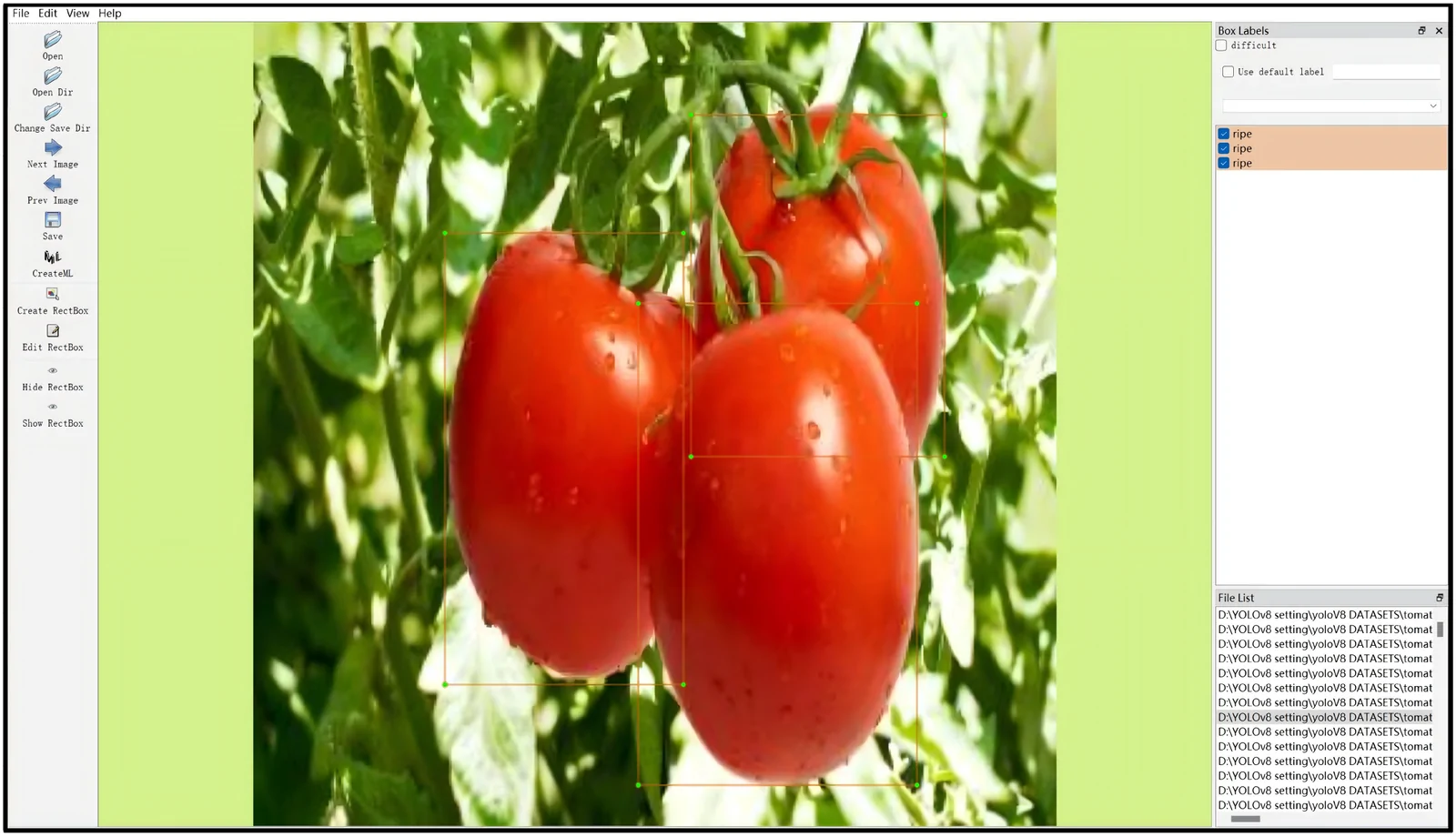
Example annotation of the cherry tomato dataset using LabelImg.

**Figure 9:**
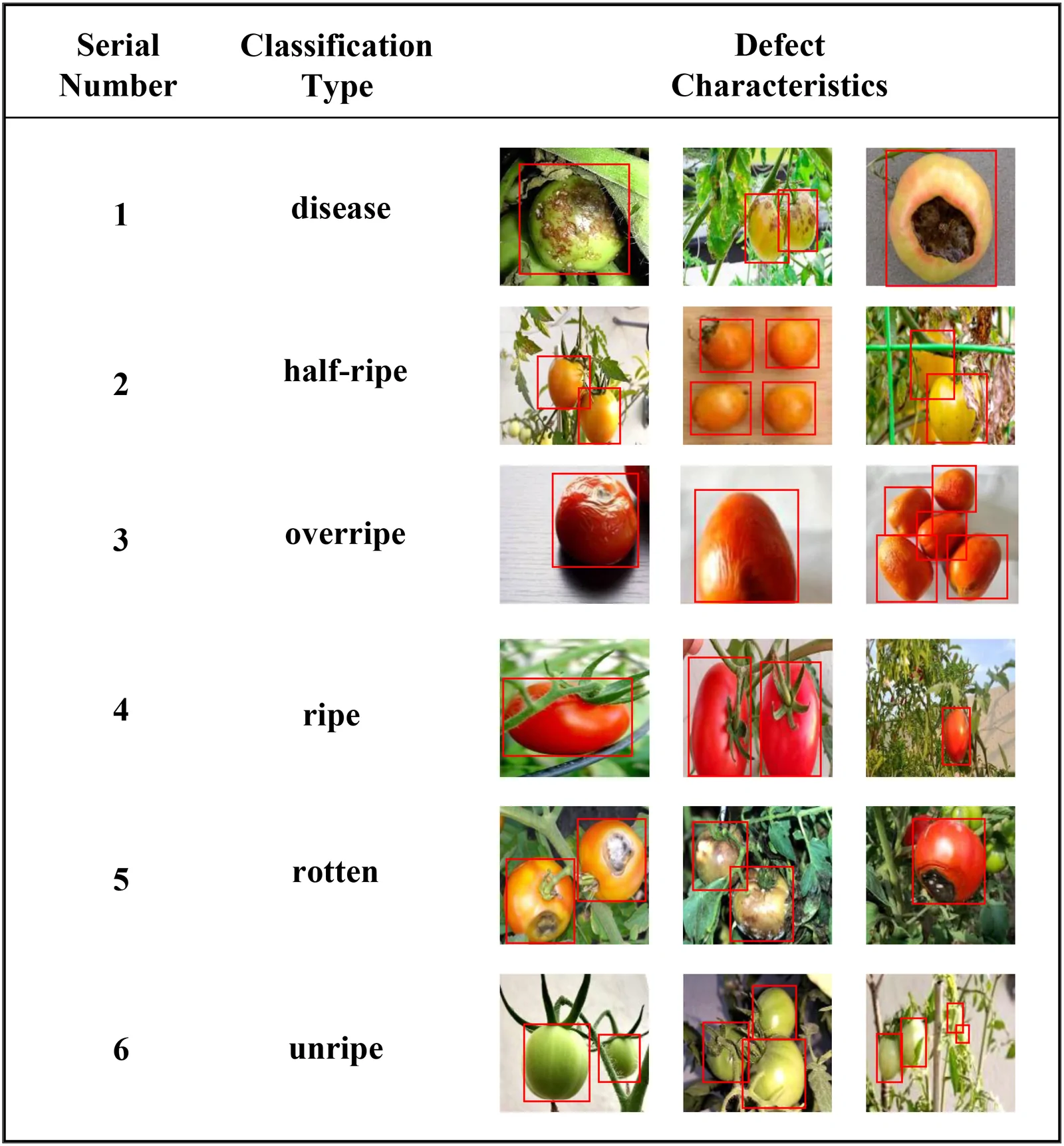
Examples of the six cherry tomato ripeness and disease categories.

To prevent data leakage between the training and evaluation subsets, the 1,583 original images were divided into training, validation, and test sets before data augmentation. The three subsets contained 1,108, 317, and 158 original images, respectively, corresponding to an approximate ratio of 7:2:1. Data augmentation was applied only to the training set, whereas the validation and test sets retained the original images for model selection and independent performance evaluation.

Four augmentation operations were used, including image enhancement, horizontal mirroring, denoising, and grayscale conversion. For each original image in the training set, four augmented images were generated. Consequently, the training set was expanded from 1,108 original images to 5,540 images, including 1,108 original images and 4,432 augmented images. The validation and test sets contained 317 and 158 unaugmented images, respectively. This partitioning and augmentation strategy ensured that transformed versions of the same source image did not appear in different subsets. The revised dataset preparation procedure is illustrated in **Figure 10**.

**Figure 10:**
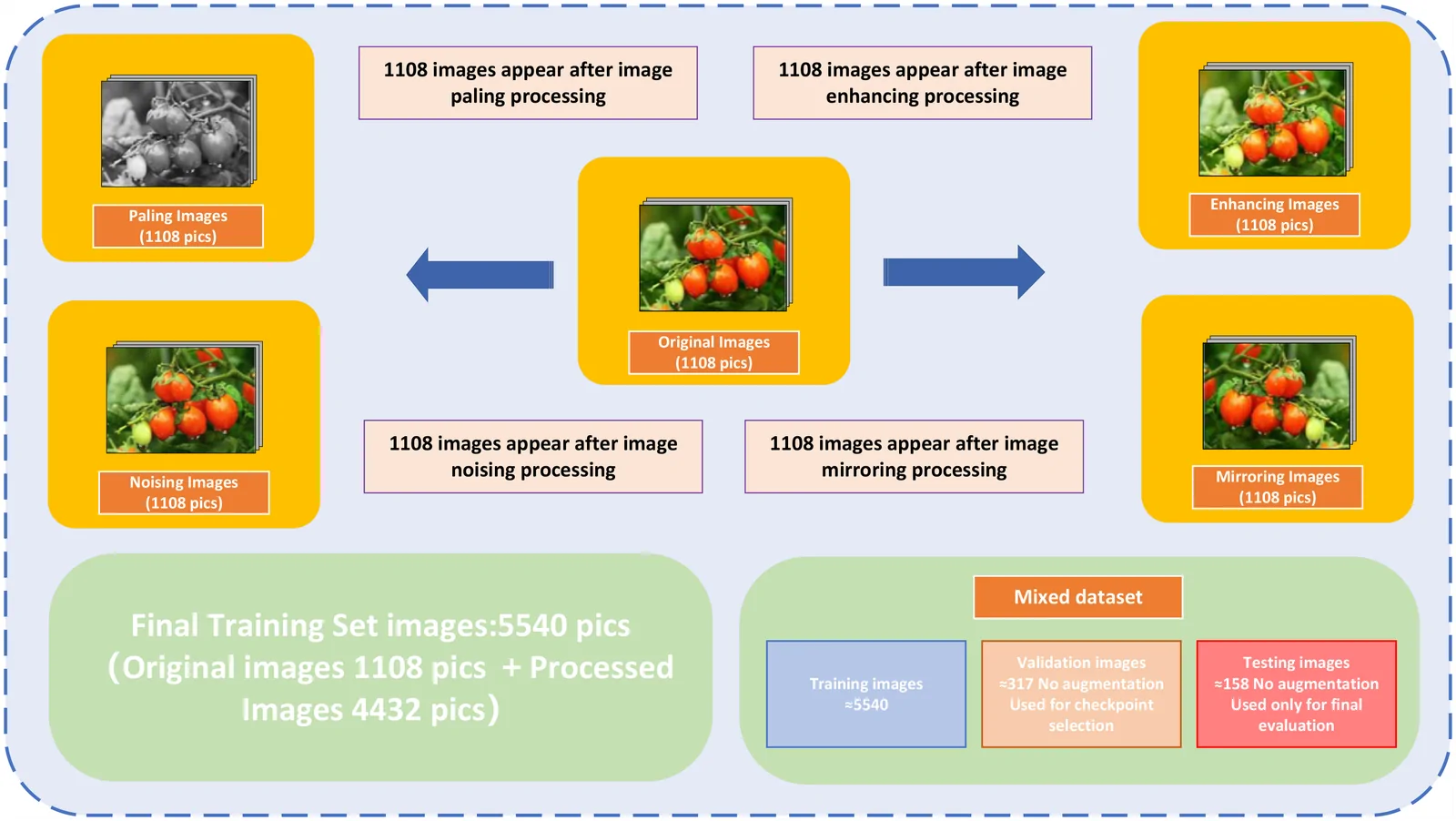
Dataset partitioning and training-set augmentation workflow.

The verified image-level distribution across the training, validation, and test subsets is summarized in **Table 2**. The dataset contains numerous annotated targets because cherry tomatoes commonly grow in dense clusters, and a single high-density image may contain several dozen visible fruits. However, the exact class-level and subset-level object-instance totals could not be independently verified with sufficient reliability from the retained annotation-summary records. To avoid implying unsupported numerical precision, the revised manuscript therefore reports only the verified image-level quantities. **Figure 8** and **9** illustrate the annotation procedure and category characteristics and should not be interpreted as representing the average target density of the complete dataset. Because a single image may contain cherry tomatoes belonging to more than one category, the sum of the category-specific image counts may exceed the number of unique images in each subset.

**Table 2:** Distribution of original images across the training, validation, and test subsets.

Class	Training	Validation	Test	Total
Disease	172	49	25	246
Half-ripe	246	70	36	352
Overripe	279	79	40	398
Ripe	501	143	72	716
Rotten	198	57	28	283
Unripe	334	95	48	477
Unique images	1,108	317	158	1,583

One image may contain targets from more than one category. Therefore, the sum of the category-specific image counts may exceed the number of unique images in each subset.

**Table 3** summarizes the verified image quantities before and after data augmentation. Data augmentation was restricted to the training subset. The validation and test subsets remained unchanged and were used for model selection and independent performance evaluation, respectively.

**Table 3:** Summary of dataset partitioning and training-set augmentation.

Subset	Original images	Augmented images	Images used
Training	1,108	4,432	5,540
Validation	317	0	317
Test	158	0	158
Total	1,583	4,432	6,015

The augmented training images retained or geometrically transformed the bounding-box annotations of their corresponding original images, as appropriate. They do not represent newly and independently annotated samples.

As shown in **Figure 9**, the dataset contains six categories representing different ripeness and disease conditions of cherry tomatoes. The category-level image distribution is reported in **Table 2**. The ripe category appears in the largest number of images, followed by the unripe and overripe categories, whereas the disease and rotten categories appear less frequently. This image-level distribution indicates a certain degree of class imbalance.

The collected and publicly selected images include close-range and distant fruits, dense clusters, fruit overlap, leaf occlusion, local shadows, complex backgrounds, and substantial target-scale variation. These conditions increase the difficulty of detecting small, partially occluded, and overlapping cherry tomatoes.

### 3.2 Network training

The experiments were conducted under Windows 11 using PyTorch 1.11.0 and NVIDIA Compute Unified Device Architecture (CUDA) 11.3. The hardware included 12 virtual central processing units (vCPUs) and an NVIDIA V100 graphics processing unit (GPU), as summarized in **Table 4**. The batch size was set to 32, the initial learning rate to 0.001, the cosine-annealing factor to 0.1, the momentum coefficient to 0.937, and the weight-decay coefficient to 0.0005. Each model was trained for 300 epochs using the Adam optimizer.

**Table 4:** Experimental environment configuration.

Category	Configuration
CPU	12 vCPUs, Intel(R) Xeon(R) Platinum 8255C CPU@ 2.50GHz
GPU	NVIDIA V100-SXM2-32GB (32GB)
System environment	Windows 11
Framework	PyTorch 1.11.0 + CUDA 11.3
Programming language	Python 3.8

Each model configuration was trained independently three times under the same dataset partition, hardware environment, and hyperparameter settings. For each run, the checkpoint with the highest validation-set mAP@0.5 was selected, and the test set was evaluated only after the checkpoint had been fixed. The reported performance values are the arithmetic means of the three independent runs. Standard deviations are reported for the principal mAP metrics included in the corresponding result tables.

**Figure 11** presents the total detection loss normalized by the training batch size rather than the unnormalized YOLOv8 loss scale. The displayed loss is defined as.

**Figure 11:**
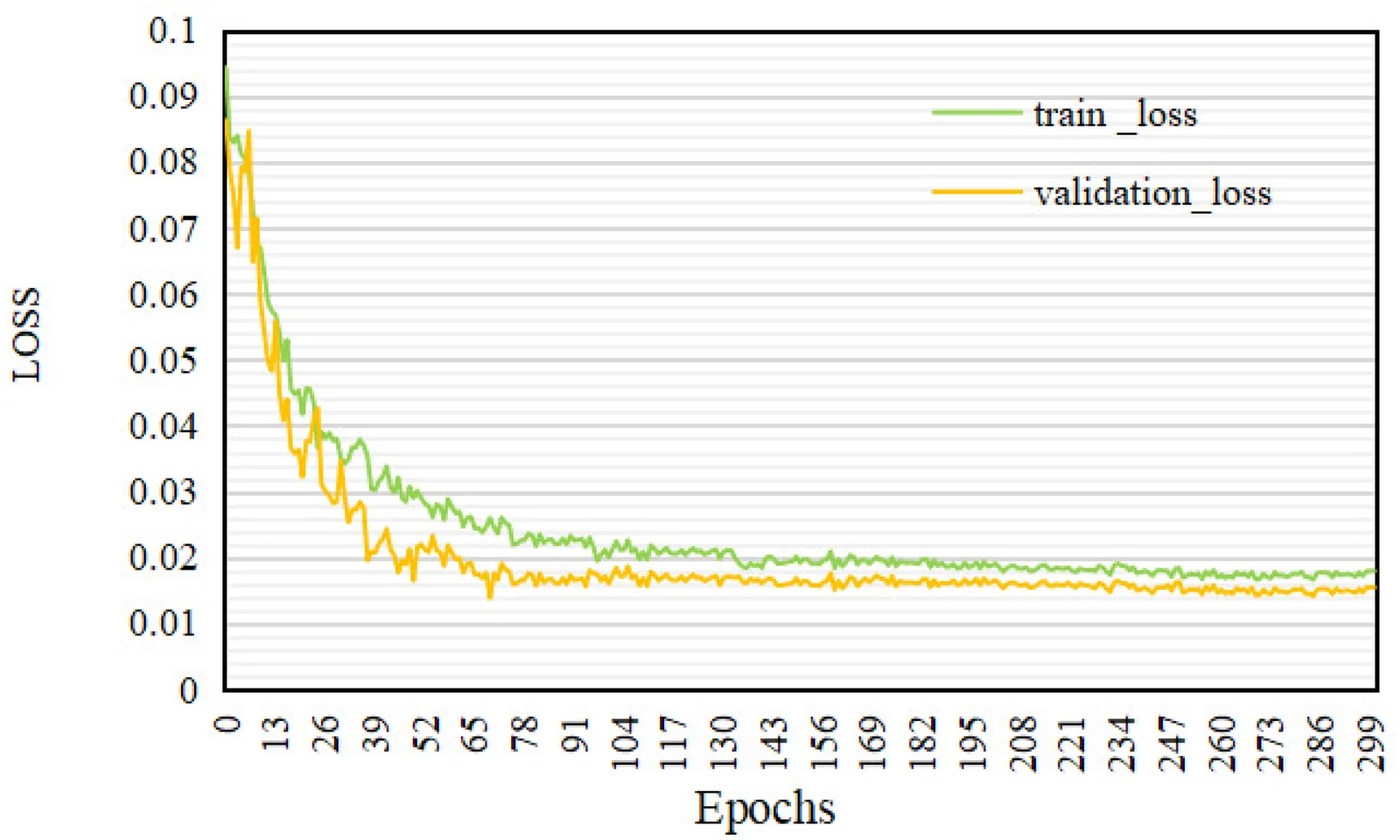
Batch-size-normalized training and validation total detection losses over 300 epochs.


$$L_{\text{norm}}=\frac{L_{\text{det}}}{B}=\frac{L_{\text{box}}+L_{\text{cls}}+L_{\text{DFL}}}{32},$$

where *L*_box_, *L*_cls_, and *L*_DFL_ denote the bounding-box regression, classification, and distribution focal loss components, respectively, and *B* = 32 is the training batch size. Consequently, the values of approximately 0.01–0.1 shown in **Figure 11** are batch-size-normalized values and should not be directly compared with unnormalized YOLOv8 total-loss values. This normalization is used only to present the loss trend consistently and does not modify the original loss used for model optimization or backpropagation.

As shown in **Figure 11**, the batch-size-normalized training and validation losses decrease rapidly during the first 100 epochs, decline more gradually between epochs 100 and 250, and subsequently approach convergence. **Figure 12** presents the normalized confusion matrix of Efficient-Tomato. The diagonal entries represent correctly classified objects, whereas the off-diagonal entries indicate interclass confusion. The predominantly high diagonal values show that Efficient-Tomato maintains strong classification performance across the six categories.

**Figure 12:**
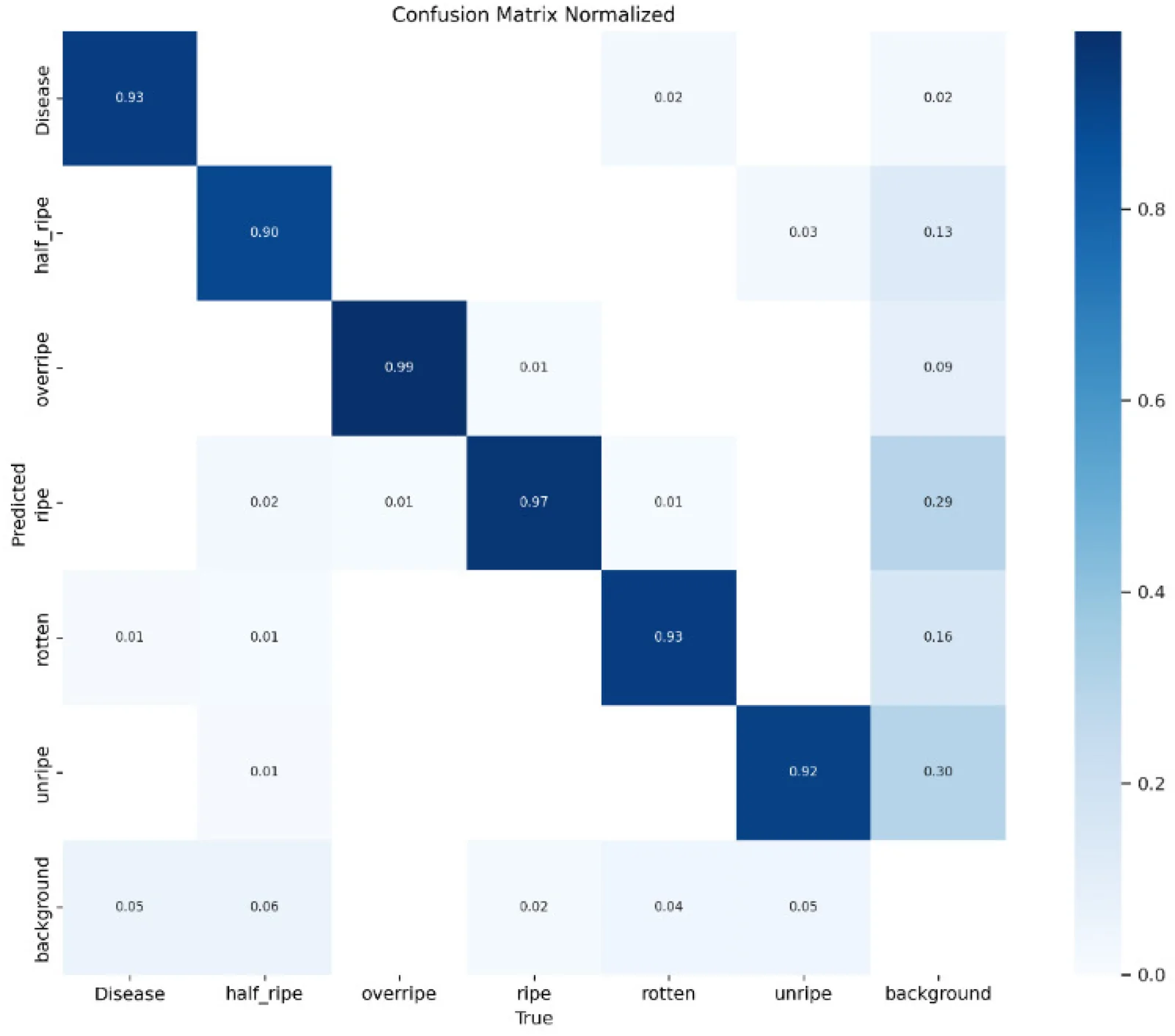
Confusion matrix of Efficient-Tomato.

### 3.3 Evaluation indicators

The performance of the detection models was evaluated using precision, recall, average precision (AP), mean average precision (mAP), F1 score, frames per second (FPS), and the number of model parameters. A prediction was counted as a true positive (TP) when its class label was correct, its confidence score was at least 0.25, and its Intersection over Union (IoU) with the matched ground-truth box was at least 0.5. A prediction with a confidence score meeting the threshold was counted as a false positive (FP) when its class label was incorrect, its IoU with the ground-truth box was below 0.5, or it was a duplicate prediction for an already matched ground-truth object. An unmatched ground-truth object was counted as a false negative (FN). Non-maximum suppression (NMS) used an IoU threshold of 0.7. The precision, recall, and F1 values reported in the result tables all use the above IoU = 0.5 matching criterion. True negatives are not explicitly used because the number of background regions is not naturally defined in object detection.

Precision measures the proportion of predicted objects that are correctly detected, whereas recall measures the proportion of ground-truth objects that are successfully detected. Their definitions are given in Equations 5, 6.

(5) $$\text{Precision}=\frac{TP}{TP+FP}$$

(6) $$\text{Recall}=\frac{TP}{TP+FN}$$

AP represents the interpolated precision averaged over 101 uniformly spaced recall levels for one category, as shown in Equation 7. The COCO-style 101-point interpolation scheme is used, where $p_{\text{interp}}\left(r\right)$ is the maximum precision obtained at any recall level $\tilde{r}\geq r$. The mAP is obtained by averaging the AP values over all *N* categories, as expressed in Equation 8. In this study, mAP@0.5 denotes AP averaged across categories at an IoU threshold of 0.5, whereas mAP@0.5:0.95 denotes AP averaged over IoU thresholds from 0.50 to 0.95 in increments of 0.05.

(7) $$\text{AP}=\frac{1}{101}\sum_{k=0}^{100}p_{\text{interp}}\left(r_{k}\right),\;r_{k}=\frac{k}{100},\;p_{\text{interp}}\left(r_{k}\right)=\underset{\tilde{r}\geq r_{k}}{\max}p\left(\tilde{r}\right)$$

(8) $$\text{mAP}=\frac{1}{N}\sum_{i=1}^{v}{\text{AP}}_{i}$$

The F1 score is the harmonic mean of precision and recall and is defined in Equation 9:

(9) $$\text{F}1=\frac{2\times \left(\text{Precision}\times \text{Recall}\right)}{\text{Precision}+\text{Recall}}$$

FPS was measured on an NVIDIA V100 GPU using an input resolution of 640×640, a batch size of 1, and FP32 precision. Before formal timing, 50 warm-up iterations were performed. The average processing time was then measured over 1,000 images, with CUDA synchronization executed immediately before and after each timed inference interval. The per-image processing time includes image resizing and normalization, model forward inference, non-maximum suppression, and detection-result post-processing, but excludes disk input, result visualization, and file saving. FPS is defined as the reciprocal of the average per-image processing time, as shown in Equation 10. A higher FPS indicates faster inference under the same hardware and measurement settings. The parameter count is additionally reported to characterize model size.

The results of the three independent runs were summarized using the arithmetic mean, and variability across runs was quantified using the sample standard deviation (SD). To maintain consistency with Equation 9, the F1 values reported in the result tables were recalculated from the corresponding tabulated mean precision and recall values. In the ablation experiment, mAP@0.5 is reported as mean ± standard deviation, consistent with the metric included in the original ablation table. In the comparative experiment, both mAP@0.5 and mAP@0.5:0.95 are reported as mean ± standard deviation.

(10) $$\text{FPS}=\frac{1}{\text{Average processing time}}$$

### 3.4 Ablation experiments

Five progressively integrated model configurations were evaluated. Each row in **Tables 5** and **6** represents a complete configuration built on the preceding row rather than an isolated single-factor experiment. Each configuration was trained independently three times under the same dataset partition, hardware environment, and hyperparameter settings. YOLOv8s served as the baseline, after which EfficientViT, BiFormer, the CARAFE integration configuration, and WIoUv1 were introduced sequentially.

**Table 5:** Ablation results averaged over three independent runs.

Model	+EfficientViT	+BiFormer	+CARAFE config.	+WIoUv1	Precision (%)	Recall (%)	mAP@0.5 (mean ± SD, %)	F1 score (%)	FPS	Parameters (M)
YOLOv8s					89.5	87.3	88.5 ± 0.7	88.4	150.2	22.5
–	✓				92.5	89.7	91.9 ± 0.3	91.1	156.9	13.2
–	✓	✓			94.4	92.8	95.1 ± 0.3	93.6	161.3	17.5
–	✓	✓	✓		95.9	96.5	96.5 ± 0.5	96.2	172.5	14.9
Efficient-Tomato	✓	✓	✓	✓	97.5	96.9	96.7 ± 0.5	97.2	172.5	14.9

Precision, recall, and mAP@0.5 are arithmetic means of three independent runs. F1 was recalculated from the tabulated mean precision and recall using Equation 9. The standard deviation is reported for mAP@0.5. Each row is a progressively integrated configuration. The CARAFE configuration includes CARAFE replacement and accompanying lightweight neck-channel adaptation. For each run, the checkpoint with the highest validation-set mAP@0.5 was evaluated on the independent test set.

**Table 6:** Mean category-level AP results of the ablation experiments across three independent runs.

Model	+EfficientViT	+BiFormer	+CARAFE config.	+WIoUv1	Disease	Half-ripe	Overripe	Ripe	Rotten	Unripe
YOLOv8s					0.852	0.847	0.973	0.909	0.863	0.868
–	✓				0.900	0.876	0.966	0.936	0.922	0.914
–	✓	✓			0.952	0.933	0.983	0.949	0.943	0.947
–	✓	✓	✓		0.973	0.940	0.991	0.973	0.966	0.944
Efficient-Tomato	✓	✓	✓	✓	0.966	0.952	0.988	0.976	0.971	0.950

All category-level AP values are arithmetic means of three independent runs. Each row is a progressively integrated configuration, and the CARAFE configuration includes the accompanying lightweight neck-channel adaptation.

The CARAFE stage requires particular clarification because it contains two coupled modifications. A controlled decomposition would consist of three matched configurations: (i) the EfficientViT+BiFormer model with the original neck channels and nearest-neighbor upsampling; (ii) the same model with only the lightweight neck-channel adaptation while retaining nearest-neighbor upsampling; and (iii) the channel-adapted model with CARAFE replacing nearest-neighbor upsampling. In such a design, the comparison between (i) and (ii) would quantify the effect of channel adaptation, whereas the comparison between (ii) and (iii) would isolate the additional effect of CARAFE under identical channel widths. The retained experimental records for the present study do not contain a separately trained channel-only configuration, so a numerical CARAFE-only gain is not reported. Accordingly, the manuscript treats the CARAFE row as an integrated configuration and avoids attributing its full accuracy or efficiency change to CARAFE alone. **Table 5** reports the mean performance of the three runs, together with the standard deviation of mAP@0.5. The category-level mean AP values are presented in **Table 6**.

Replacing the YOLOv8s backbone with EfficientViT increased the mean precision from 89.5% to 92.5%, recall from 87.3% to 89.7%, mAP@0.5 from 88.5% to 91.9%, and the recalculated F1 score from 88.4% to 91.1%. These changes correspond to gains of 3.0, 2.4, 3.4, and 2.7 percentage points, respectively. FPS increased by approximately 4.5%, while the parameter count decreased by 41.3%. These results indicate that EfficientViT provides a substantially lighter feature-extraction backbone while improving the principal detection metrics.

Adding BiFormer to the EfficientViT-based configuration further increased mean precision, recall, mAP@0.5, and the recalculated F1 score by 1.9, 3.1, 3.2, and 2.5 percentage points, respectively. The parameter count rose from 13.2 M to 17.5 M, indicating additional model complexity. The measured FPS on the NVIDIA V100 GPU changed slightly from 156.9 to 161.3; however, inference speed is also affected by operator implementation, memory access, and hardware parallelism, so this variation is not attributed directly to BiFormer. The primary effect of BiFormer is interpreted as an improvement in spatial and contextual feature representation, particularly for small and occluded objects.

The integrated channel-adaptation+CARAFE configuration increased mean precision by 1.5 percentage points, recall by 3.7 percentage points, mAP@0.5 by 1.4 percentage points, and the recalculated F1 score by 2.6 percentage points relative to the preceding EfficientViT+BiFormer configuration. Its parameter count decreased from 17.5 M to 14.9 M, while the measured GPU throughput changed from 161.3 to 172.5 FPS. The reduction in parameter count is associated with the lightweight neck-channel adaptation offsetting the additional parameters introduced by content-aware upsampling; it should not be interpreted as a parameter reduction caused by CARAFE itself. Likewise, because a channel-only configuration was not independently trained, the observed accuracy changes cannot be decomposed quantitatively into a channel-width effect and a CARAFE effect. The result therefore supports the effectiveness of the integrated neck redesign, while the independent contribution of CARAFE remains to be established by a matched channel-only control.

The final configuration incorporated WIoUv1 into the bounding-box regression loss. Relative to the preceding configuration, mean precision increased by 1.6 percentage points, recall by 0.4 percentage points, mAP@0.5 by 0.2 percentage points, and the recalculated F1 score by 1.0 percentage point. Across the three independent runs, Efficient-Tomato achieved mean values of 97.5% precision, 96.9% recall, and 96.7% mAP@0.5, with a corresponding F1 score of 97.2%. Because WIoUv1 is a training-stage loss without learnable parameters and does not change the inference network, the parameter count and FPS remain 14.9 M and 172.5, respectively, before and after its introduction. Compared with YOLOv8s, the complete model increased precision, recall, mAP@0.5, and F1 score by 8.0, 9.6, 8.2, and 8.8 percentage points, respectively, while increasing FPS by 14.8% and reducing the parameter count by approximately 33.8%. The run-to-run variability of mAP@0.5 is reported in **Table 5**.

The category-level results in **Table 6** further demonstrate that the components have complementary effects. The final model achieved its highest AP for overripe and ripe targets, while disease and overripe AP showed small fluctuations after the introduction of WIoUv1. Therefore, the final improvement should be interpreted as an increase in overall detection performance rather than a uniform gain for every category. The precision–recall (PR) and F1 curves in **Figures 13** and **14** provide additional comparisons between Efficient-Tomato and YOLOv8s.

**Figure 13:**
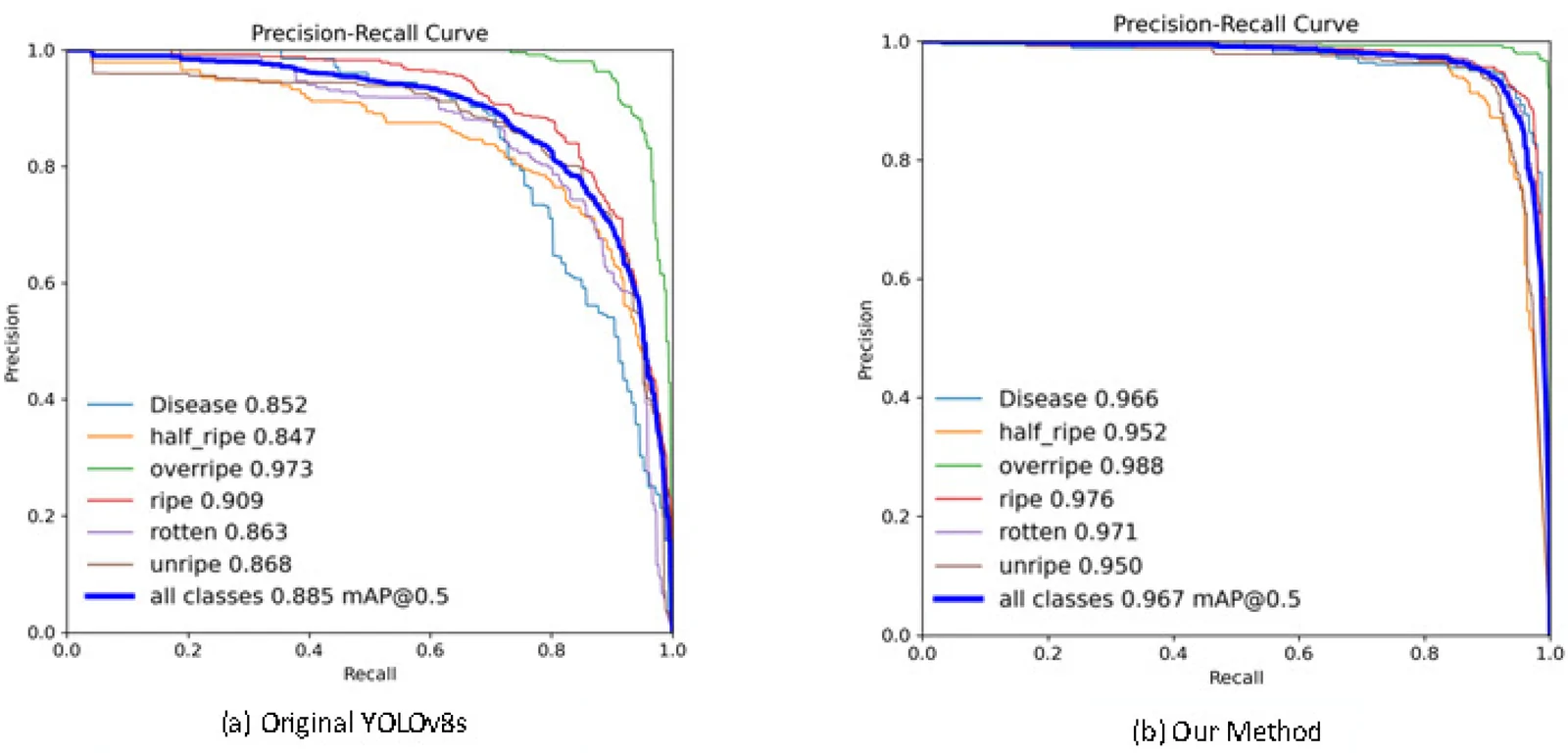
Precision-recall (PR) curve comparison: **(a)** original YOLOv8s; **(b)** Efficient-Toma.

**Figure 14:**
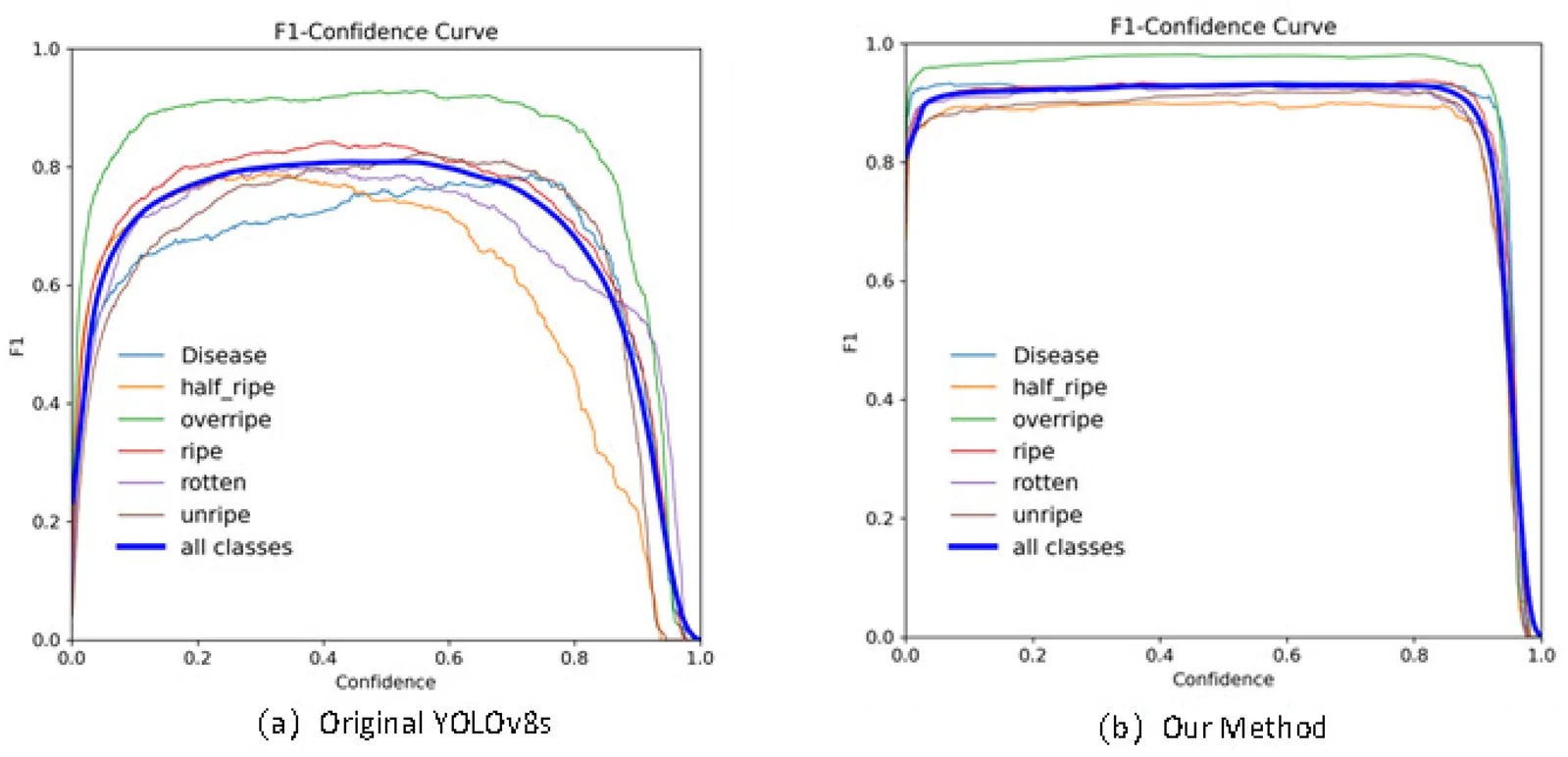
F1-confidence curve comparison: **(a)** original YOLOv8s; **(b)** Efficient-Tomato.

**Figure 15** compares the two models under different visual conditions. In Scenes 1–3, YOLOv8s exhibits missed detections for distant or partially occluded targets and false positives when unripe fruits have colors similar to surrounding leaves. Efficient-Tomato detects more of these challenging targets with higher confidence. In Scenes 4 and 5, both models identify the major objects, but Efficient-Tomato provides more stable predictions. These qualitative results are consistent with the quantitative improvements in recall and F1 score.

**Figure 15:**
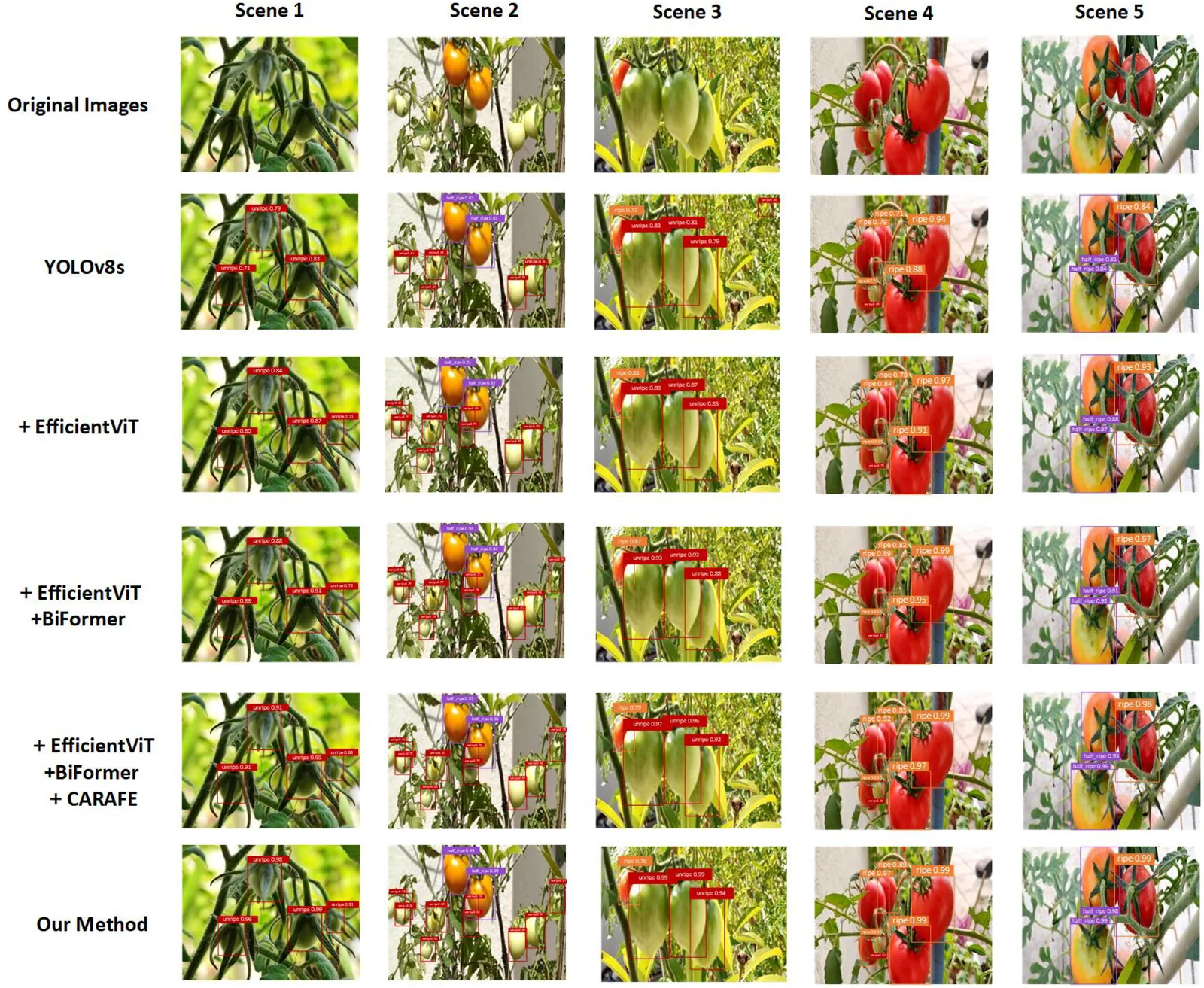
Detection comparison between YOLOv8s and Efficient-Tomato under multiple scenarios.

### 3.5 Comparative experiments

Efficient-Tomato was compared with SSD, YOLOv5s, YOLO-Tomato, and YOLOv8s using the same dataset partition, evaluation protocol, and hardware environment. Each model was trained independently three times. The comparison considered model size, inference speed, precision, recall, mAP@0.5, mAP@0.5:0.95, F1 score, and category-level AP. **Figure 16** presents representative mAP@0.5 curves over 300 training epochs. All models improve rapidly during the early training stage and gradually converge after approximately 250 epochs. Efficient-Tomato reaches a mean mAP@0.5 of 96.7%, exceeding the corresponding mean values of the comparison models.

**Figure 16:**
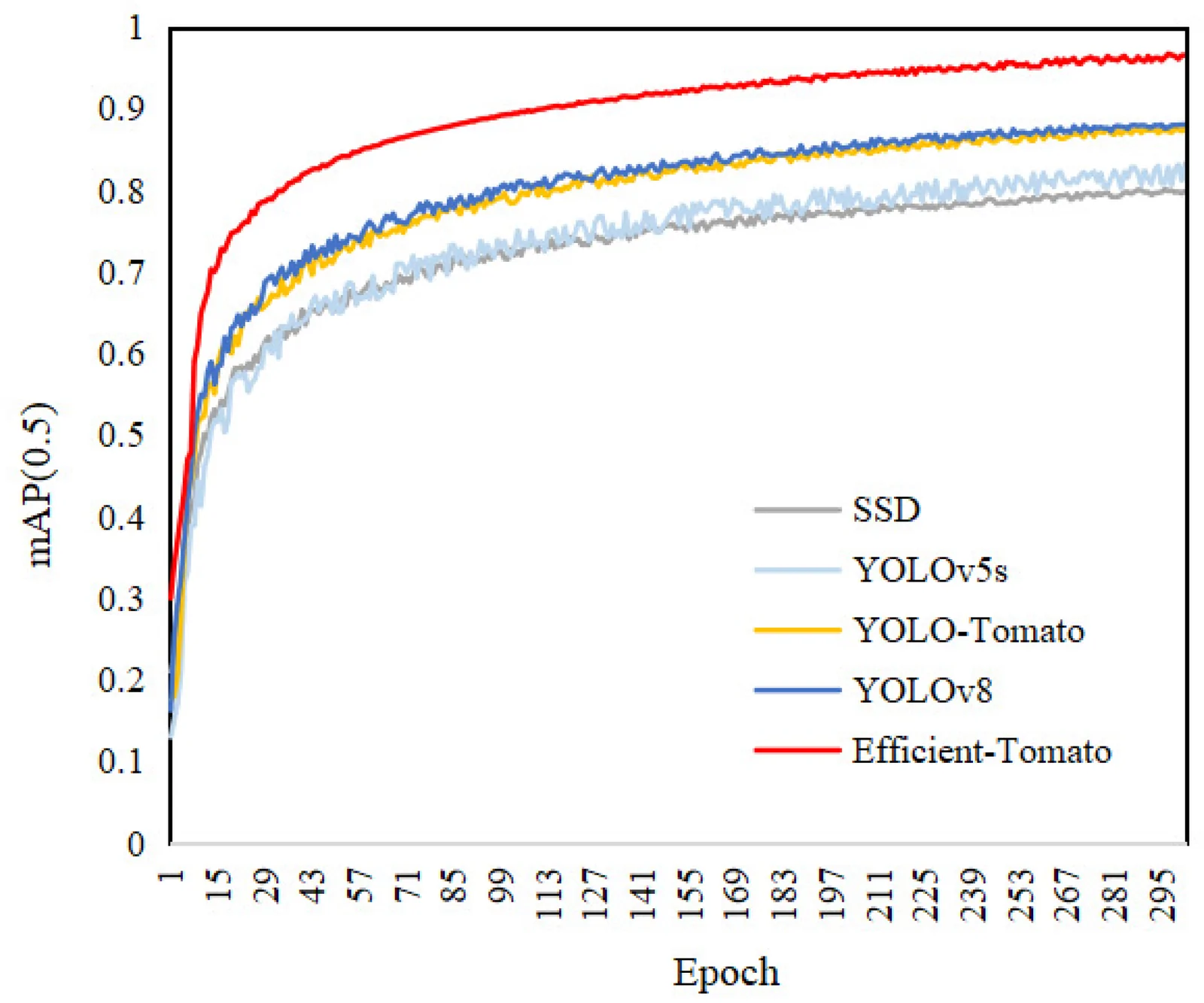
mAP@0.5 curves of the compared detection models during training.

For each independent run, the checkpoint achieving the highest mAP@0.5 on the validation set was selected. The test set was not used for model selection or hyperparameter tuning and was evaluated only after the checkpoint had been fixed. **Tables 7** and **8** report the mean results of the three runs. Standard deviations are additionally provided for mAP@0.5 and mAP@0.5:0.95 in **Table 7**.

**Table 7:** Comparison of detection methods averaged over three independent runs.

Model	Parameters (M)	FPS	mAP@0.5 (mean ± SD, %)	mAP@0.5:0.95 (mean ± SD, %)	Precision (%)	Recall (%)	F1 score (%)
SSD	91.6	22	80.3 ± 0.6	74.9 ± 0.5	78.9	82.1	80.5
YOLOv5s	11.3	92	83.2 ± 0.5	76.5 ± 0.6	81.6	79.9	80.7
YOLO-Tomato	17.6	109	87.6 ± 0.7	80.1 ± 0.7	87.5	87.3	87.4
YOLOv8s	22.5	150.2	88.5 ± 0.7	81.6 ± 0.6	89.5	87.3	88.4
Efficient-Tomato	14.9	172.5	96.7 ± 0.5	87.9 ± 0.1	97.5	96.9	97.2

Precision, recall, and the two mAP values are arithmetic means of three independent runs. F1 was recalculated from the tabulated mean precision and recall using Equation 9. Standard deviations are reported for mAP@0.5 and mAP@0.5:0.95. For the same model, the mAP@0.5 mean and standard deviation are kept consistent with the ablation table. For each run, the checkpoint with the highest validation-set mAP@0.5 was evaluated on the independent test set.

**Table 8:** Mean category-level AP comparison of different detection methods across three independent runs.

Model	Disease	Half-ripe	Overripe	Ripe	Rotten	Unripe
SSD	0.821	0.795	0.813	0.802	0.794	0.793
YOLOv5s	0.831	0.805	0.826	0.803	0.827	0.901
YOLO-Tomato	0.879	0.859	0.871	0.889	0.821	0.937
YOLOv8s	0.852	0.847	0.973	0.909	0.863	0.868
Efficient-Tomato	0.966	0.952	0.988	0.976	0.971	0.950

All category-level AP values are arithmetic means of three independent runs.

Compared with SSD, Efficient-Tomato increased mean precision, recall, mAP@0.5, mAP@0.5:0.95, and the recalculated F1 score by 18.6, 14.8, 16.4, 13.0, and 16.7 percentage points, respectively, while reducing the parameter count by approximately 83.7%. The category-level AP gains ranged from 14.5 to 17.7 percentage points.

Compared with YOLOv5s, Efficient-Tomato increased mean precision by 15.9 percentage points, recall by 17.0 percentage points, mAP@0.5 by 13.5 percentage points, mAP@0.5:0.95 by 11.4 percentage points, and the recalculated F1 score by 16.5 percentage points. Efficient-Tomato used more parameters than YOLOv5s but achieved higher FPS and detection performance, representing a different trade-off between model size and accuracy.

Compared with YOLO-Tomato, Efficient-Tomato increased mean precision, recall, mAP@0.5, mAP@0.5:0.95, and the recalculated F1 score by 10.0, 9.6, 9.1, 7.8, and 9.8 percentage points, respectively. The parameter count decreased by approximately 15.3%, while FPS increased by approximately 58.3%.

Compared with YOLOv8s, Efficient-Tomato increased mean precision and recall by 8.0 and 9.6 percentage points, respectively. The mean mAP@0.5 increased by 8.2 percentage points, mAP@0.5:0.95 by 6.3 percentage points, and the recalculated F1 score by 8.8 percentage points. Meanwhile, FPS increased by 14.8%, and the parameter count decreased by approximately 33.8%. The mean AP gains for disease, half-ripe, overripe, ripe, rotten, and unripe targets were 11.4, 10.5, 1.5, 6.7, 10.8, and 8.2 percentage points, respectively.

The visual comparisons in **Figure 17** further illustrate the differences among the models. In Scenes 1 and 2, all models detect the major cherry tomatoes, but Efficient-Tomato produces more stable predictions and higher confidence. In Scene 3, the color similarity between unripe fruits and leaves causes false positives or missed detections in SSD, YOLOv5s, YOLO-Tomato, and YOLOv8s, whereas Efficient-Tomato detects the target more reliably. In Scenes 4 and 5, which contain numerous overlapping and partially occluded fruits, the comparison models show more missed detections and false positives. Efficient-Tomato provides more complete detections, supporting the complementary effects of selective feature enhancement, content-aware fusion, and optimized localization.

**Figure 17:**
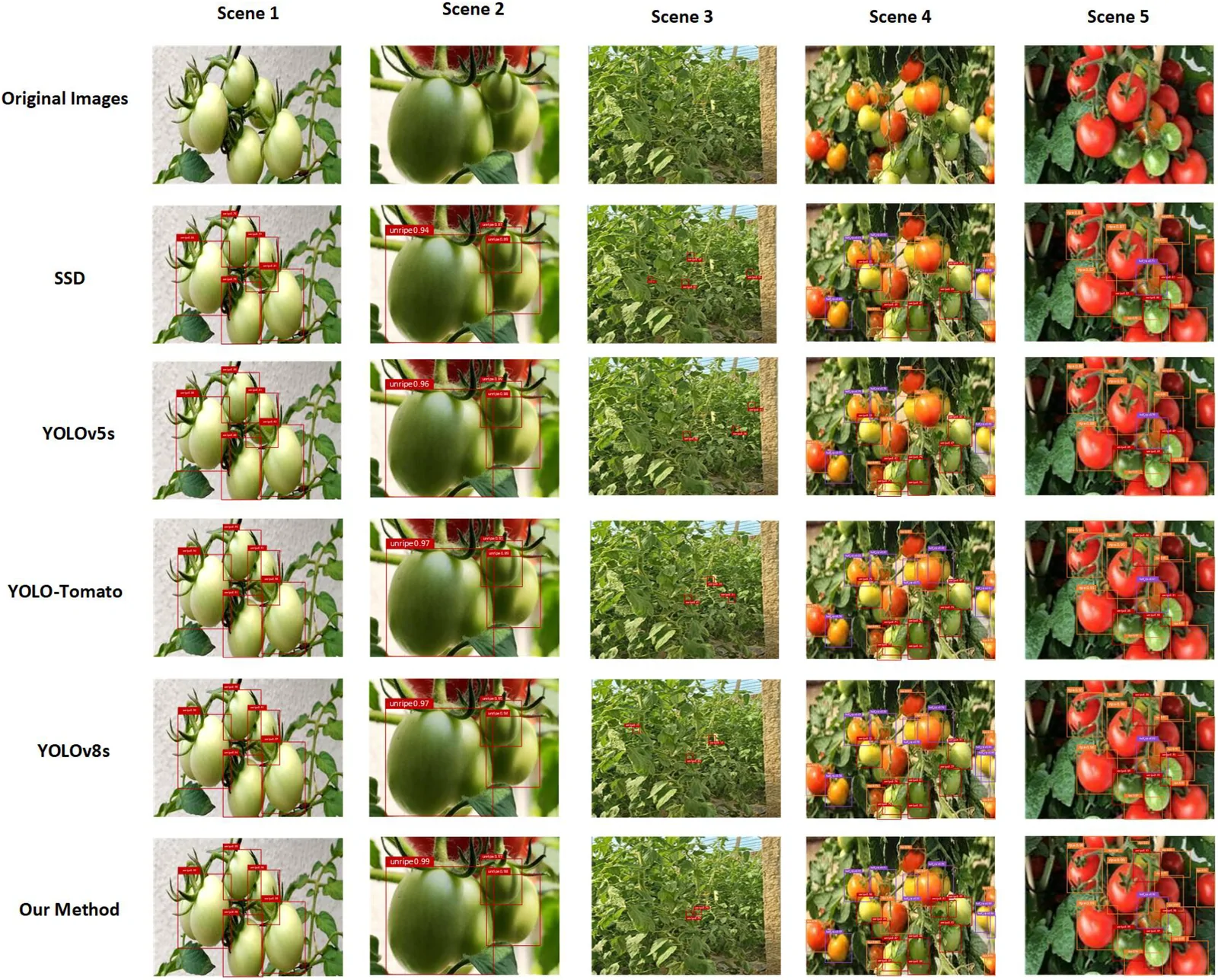
Detection results of different models under multiple cherry tomato scenarios.

Overall, Efficient-Tomato achieves the best aggregate detection performance among the compared models while maintaining a moderate parameter count and favorable throughput on the NVIDIA V100 GPU used in this study. These results support the effectiveness of the task-oriented coordinated design under the reported hardware and timing protocol. However, the FPS values are hardware-specific and should not be interpreted as evidence of real-time performance on embedded processors, edge accelerators, or operational harvesting robots.

### 3.6 External generalization validation

To further evaluate the generalization ability of the trained detector beyond the original mixed-source dataset, an additional independent public dataset, the Tomato Ripeness 2 Dataset from Roboflow Universe (2025), was introduced for external validation. This dataset was not used during model training, validation, checkpoint selection, or hyperparameter tuning. Instead, the final Efficient-Tomato model trained on the original dataset was directly applied to the external images without retraining or fine-tuning. Because the external dataset differs from the original data in image source, background, shooting conditions, target scale, and visual appearance, this evaluation provides a cross-dataset test of the model’s robustness to domain variation.

The external dataset contains ripeness-related categories that partially overlap semantically with the categories used in this study. Therefore, the external evaluation was conducted only on categories with clear semantic correspondence between the two datasets, rather than forcing unmatched labels into the six-category taxonomy used for model development. This design avoids introducing artificial label equivalence and provides a more conservative assessment of cross-domain generalization. Representative detection results on the external dataset are presented separately to qualitatively evaluate the cross-dataset generalization ability of the trained Efficient-Tomato model.

### 3.7 Limitations

Although Efficient-Tomato achieved favorable results on the current dataset and was additionally evaluated on an independent external tomato-ripeness dataset, several limitations remain. First, the field-collected portion of the development dataset was mainly obtained from a limited number of production environments, and the external validation dataset does not cover all six categories used in this study. Therefore, broader multi-site and multi-season validation across different cultivars, camera devices, and geographical locations is still needed. Second, extremely severe occlusion, strong backlighting, motion blur, and color similarity between unripe fruits and leaves may still lead to missed or incorrect detections. Third, the current progressive ablation does not include a separately trained channel-only neck configuration. Consequently, the independent effects of lightweight channel adaptation and CARAFE cannot be numerically disentangled from the present records, and the corresponding claims are deliberately restricted to the integrated neck configuration. A future controlled ablation will compare the original-channel+nearest-neighbor, adapted-channel+nearest-neighbor, and adapted-channel+CARAFE settings under otherwise identical conditions. Fourth, the current inference-speed evaluation was conducted on an NVIDIA V100 GPU rather than on an embedded device or a harvesting robot. The reported FPS therefore characterizes GPU-side computational efficiency under the specified protocol and does not establish real-time performance or field-deployment readiness on agricultural edge hardware. Future work will include broader cross-domain validation, additional field data, controlled neck-component ablation, model quantization, embedded-device testing, and integration with robotic localization and grasping systems.

## 4 Conclusion

This study presented Efficient-Tomato, a lightweight YOLOv8s-based framework for detecting six cherry tomato ripeness and disease categories: disease, half-ripe, overripe, ripe, rotten, and unripe. The contribution of the framework lies in the task-oriented coordination of complementary components rather than in proposing a new individual operator. EfficientViT provides lightweight local and global feature extraction; BiFormer strengthens selective spatial and contextual representation; CARAFE improves content-aware multiscale feature reconstruction; and WIoUv1 refines bounding-box regression while DFL is retained.

Across three independent runs on the current test set, Efficient-Tomato achieved mean values of 97.5% precision, 96.9% recall, 96.7% mAP@0.5, and 87.9% mAP@0.5:0.95, with an F1 score of 97.2% recalculated from the tabulated mean precision and recall. Relative to YOLOv8s, these values represent gains of 8.0, 9.6, 8.2, 6.3, and 8.8 percentage points, respectively. FPS increased by 14.8%, while the parameter count decreased by approximately 33.8%. The ablation results indicate that the four components contribute at different stages of the detection pipeline and collectively improve the balance between feature representation, small-target reconstruction, localization, and computational efficiency.

Several limitations remain. Although an independent external tomato-ripeness dataset was introduced to provide an additional cross-dataset assessment, the development data were still obtained from a limited number of field and open-source environments, and the external dataset does not cover all six categories considered in this study. Broader validation across cultivars, seasons, camera devices, and geographical locations is therefore still required. Severe occlusion, strong backlighting, motion blur, and domain shifts may still reduce performance. In addition, the present progressive ablation does not isolate a channel-only neck configuration, so the effect of CARAFE cannot yet be separated quantitatively from the accompanying lightweight channel adaptation. Inference speed was also evaluated only on an NVIDIA V100 GPU rather than on embedded agricultural hardware. Therefore, the current FPS results should be interpreted as hardware-specific computational measurements rather than evidence of real-time robotic deployment. Future work will focus on broader cross-domain evaluation, controlled channel-only versus channel+CARAFE ablation, additional field data, model quantization, embedded-device testing, and integration with robotic localization and grasping systems.

## Data Availability

The field-collected images used in this study are subject to restrictions under the cooperation agreement with the participating vegetable production base and may be available from the corresponding author upon reasonable request and with permission from the data provider. The open-source images were obtained from the Roboflow platform and remain subject to the license and access conditions of the corresponding dataset source.
